# Role and mechanism of molecular hydrogen in the treatment of Parkinson’s diseases

**DOI:** 10.3389/fnins.2025.1576773

**Published:** 2025-04-23

**Authors:** Fengjiao Wang, Guangjie Zhang, Qingfeng Zhai

**Affiliations:** ^1^School of Public Health, Shandong Second Medical University, Weifang, China; ^2^Department of Medical Technology and Nursing, Laiwu Vocational and Technical College, Jinan, China

**Keywords:** Parkinson’s disease, hydrogen, neuroprotection, oxidative stress, neurological inflammation

## Abstract

Parkinson’s disease (PD) is a complex neurodegenerative disorder characterized by a pathology that includes the aggregation of alpha-synuclein (α-syn), oxidative stress, and neuroinflammation. While existing treatments can alleviate motor symptoms, they have limited efficacy in slowing disease progression and improving non-motor symptoms. In recent years, molecular hydrogen has been recognized for its potential neuroprotective effects, attributed to its selective antioxidant and anti-inflammatory properties. While preclinical studies demonstrate promising results, clinical trials conducted thus far have yielded mixed outcomes, with some trials reporting limited or no therapeutic benefit. This review systematically analyzes the mechanisms of action of molecular hydrogen in PD and related neurodegenerative disorders, emphasizing its antioxidant, anti-inflammatory, and anti-apoptotic properties. By evaluating evidence from both preclinical and clinical studies, this paper explores the potential of molecular hydrogen to attenuate oxidative stress, modulate inflammatory responses, and inhibit apoptosis in neuronal cells, while also identifying key gaps in current research. As a novel neuroprotective agent, molecular hydrogen holds potential in PD and other neurodegenerative diseases, but further well-designed clinical trials are needed to validate its efficacy. Future studies should focus on elucidating the mechanisms through which hydrogen exerts its neuroprotective effects, particularly concerning α-syn aggregation and its clearance pathways, as well as Nrf2-mediated immunomodulation. Furthermore, large-scale, multicenter clinical trials are necessary to establish efficacy benchmarks and personalized delivery protocols.

## 1 Introduction

Parkinson’s disease (PD) is a complex neurodegenerative disorder influenced by genetic, environmental, oxidative stress, and neuroinflammatory factors. A key aspect of its pathology is the aggregation of alpha-synuclein (α-syn), which disrupts cellular homeostasis and promotes neuronal death through oxidative damage and microglial activation. Recent studies suggest that PD pathology may originate in the gut. Gut dysbiosis and reactive oxygen species (ROS)-induced oxidative stress contribute to the accumulation of α-syn, which subsequently propagates to the brain via the vagus nerve, thereby establishing a gut-brain axis mechanism in disease progression (Soni et al., 2024). Current treatment methods for PD primarily focus on symptom management, including pharmacological therapy, surgical intervention, and adjunctive therapies. Although pharmacological therapy has demonstrated significant efficacy in improving motor symptoms, its effectiveness often diminishes as the disease progresses and is accompanied by severe side effects. While surgical intervention can be effective for some patients, its applicability is limited, and it cannot halt the ongoing progression of the disease. Therefore, existing treatment methods still fail to adequately meet the clinical demand for slowing disease progression and improving non-motor symptoms. In recent years, antioxidant-based strategies have garnered considerable attention, particularly those that target endogenous antioxidant pathways, such as the GSK-3β-mediated regulation of the nuclear factor erythroid 2-related factor 2 (Nrf2)/heme oxygenase 1 (HO-1) axis. This approach demonstrates strong potential for neuroprotection in PD by alleviating oxidative damage and preserving dopaminergic neurons (Soni and Kumar, 2022).

Emerging research underscores the potential of molecular hydrogen as a neuroprotective agent, utilizing its selective antioxidant and anti-inflammatory properties. By neutralizing free radicals and modulating apoptotic pathways, molecular hydrogen presents a promising strategy to combat multifactorial neurodegeneration. Meanwhile, the multifaceted roles of nutraceuticals, including polyphenols, vitamins, and the Mediterranean diet, in modulating oxidative stress, neuroinflammation, and mitochondrial dysfunction have been extensively studied (Soni et al., 2024). However, while preclinical studies have shown positive effects, clinical trials have thus far produced mixed results, with some studies failing to demonstrate a clear therapeutic benefit in patients with PD. These inconsistent results suggest that while hydrogen may have potential as a neuroprotective agent, its efficacy in clinical settings remains to be definitively proven. This review assesses its mechanisms and clinical evidence, while identifying critical research gaps to inform future therapeutic strategies for PD.

## 2 PD pathogenic network

### 2.1 Overview of PD

PD is the second most common neurodegenerative disorder after Alzheimer’s disease. It was first described by James Parkinson in 1817 (Cordeiro et al., 2024). Epidemiological data indicate that approximately 7 million people worldwide are affected by PD, a figure projected to rise to 12 million by 2040 (Tichelaar et al., 2023). The hallmark clinical features of PD include resting tremor, muscle rigidity, bradykinesia, and postural instability, all of which significantly impact patients’ quality of life (Heikkila et al., 1989). These clinical manifestations are closely linked to the underlying pathological processes, including the progressive loss of dopaminergic neurons in the substantia nigra and the formation of Lewy bodies (LB). LB, which primarily consist of α-syn, ubiquitin, and other proteins, are hallmark pathological markers of PD. The definitive loss of dopaminergic neurons disrupts the basal ganglia circuitry, thereby eliciting the motor symptoms associated with PD (Nagatsu et al., 2000). Furthermore, the abnormal aggregation of α-syn to form LB interferes with cellular autophagy and protein degradation pathways, representing a significant indicator of neuronal degeneration and cell death (Meredith et al., 2002). Recent studies have also highlighted the role of α-syn propagation in spreading pathology across brain regions, further complicating the disease progression (Shin et al., 2023). Despite significant advances in understanding PD pathogenesis, effective disease-modifying therapies remain elusive, underscoring the need for further research into the molecular mechanisms underlying the disease.

### 2.2 Genetic factors

Research on the genetic mechanisms underlying PD indicates that its onset can be attributed to single-gene mutations or the interaction of multiple genes with environmental factors. In the context of monogenic inheritance, the Leucine-rich repeat kinase 2 (LRRK2) gene is a prevalent pathogenic gene, with the Gly2019Ser mutation frequently observed in European, North African, and Jewish populations. In contrast, the Asn1437Asp and Ile2020Thr mutations are more predominant in Chinese and Japanese populations, respectively (Kluss et al., 2019). Mutations or copy number variations in the Synuclein Aggregation Compound (SNCA) gene can enhance the expression of α-syn or facilitate its abnormal aggregation, serving as a core driving factor in the development of PD. Patients harboring SNCA mutations often present a rapidly progressive disease course, with pathological features that overlap with those of multiple system atrophy and Lewy body dementia. However, SNCA mutations represent a rare etiology of autosomal dominant PD (Morris et al., 2024). Furthermore, mutations in the autosomal recessive genes Parkin (PRKN), PTEN-induced kinase 1 (PINK1), and Parkinsonism associated deglycase 1 (DJ-1) contribute to the pathogenesis of autosomal recessive PD (Di Rita et al., 2018). Genome-wide association studies (GWAS) have elucidated the polygenic risk architecture of PD, identifying over 90 genetic loci associated with disease susceptibility to date. Among these, the SNCA and MAPT loci demonstrate the strongest associations in European populations, while the GBA1 locus emerges as the principal risk gene in African populations (Rizig et al., 2023). Multiple risk loci are enriched in lysosomal function and immune-inflammatory pathways, indicating that genetic background contributes to disease onset by disrupting protein homeostasis and the immune microenvironment.

### 2.3 α-syn-driven pathogenic cascade

#### 2.3.1 α-syn aggregation and mitochondrial dysfunction

α-syn is a protein that is highly expressed in the central nervous system, comprising approximately 1% of the total neuronal protein (Haque et al., 2022; Bernal-Conde et al., 2019; Fouke et al., 2021). Under pathological conditions such as oxidative stress, genetic mutations (e.g., SNCA gene amplification or point mutations), or impaired proteasome function, α-syn forms oligomers through liquid–liquid phase separation (LLPS) (Sawner et al., 2021; Ray et al., 2020), which eventually develop into LB (Mahul-Mellier et al., 2020). Furthermore, pathological α-syn spreads between neurons via a prion-like mechanism, inducing the misfolding of normal α-syn proteins, thereby accelerating degenerative changes in dopaminergic neurons (Liu et al., 2022).

The abnormal aggregation of *α-syn* can disrupt mitochondrial homeostasis through several mechanisms: (1) Inhibition of mitochondrial membrane fusion: *In vitro* experiments have demonstrated that the direct interaction of α-syn with the mitochondrial membrane can lead to mitochondrial fragmentation and activation of the apoptotic pathway (Panicker et al., 2021). (2) Interference with mitochondrial fusion protein function: Oligomeric α-syn binds to the lipid components of the outer mitochondrial membrane (OMM), thereby reducing the rate of mitochondrial fusion and resulting in mitochondrial fragmentation (Panicker et al., 2021; Pozo Devoto and Falzone, 2017). (3) Disruption of membrane integrity: α-syn interacts with the Translocase of the Outer Mitochondrial Membrane (TOM) complex (e.g., TOM40, TOM20) and the Voltage-Dependent Anion-Selective Channel 1 (VDAC1), which leads to abnormal membrane permeability (McCarthy et al., 2022). (4) Inhibition of the electron transport chain: α-syn aggregation impedes ATP production and increases the generation of ROS by diminishing the activity of mitochondrial complex I (Indrieri et al., 2020; Wang et al., 2020). The inhibition of complex I activity can further promote the abnormal accumulation of α-syn, creating a vicious cycle (Betarbet et al., 2000).

PINK1/Parkin-mediated mitophagy is a critical mechanism for the clearance of damaged mitochondria. However, overexpression of α-syn interferes with this process. *α-syn* competitively binds to TOM20, which hinders the localization of PINK1 on the outer mitochondrial membrane, thereby inhibiting the recruitment of Parkin and the ubiquitination of mitochondria (Arena et al., 2013). This disruption leads to the persistent accumulation of dysfunctional mitochondria, exacerbating oxidative stress and causing an energy crisis. Furthermore, the overexpression of α-syn disrupts the interaction between mitochondria and the endoplasmic reticulum, interfering with Ca^2+^ homeostasis and further inducing defects in mitochondrial fission (Tassone et al., 2023).

**Figure 1:**
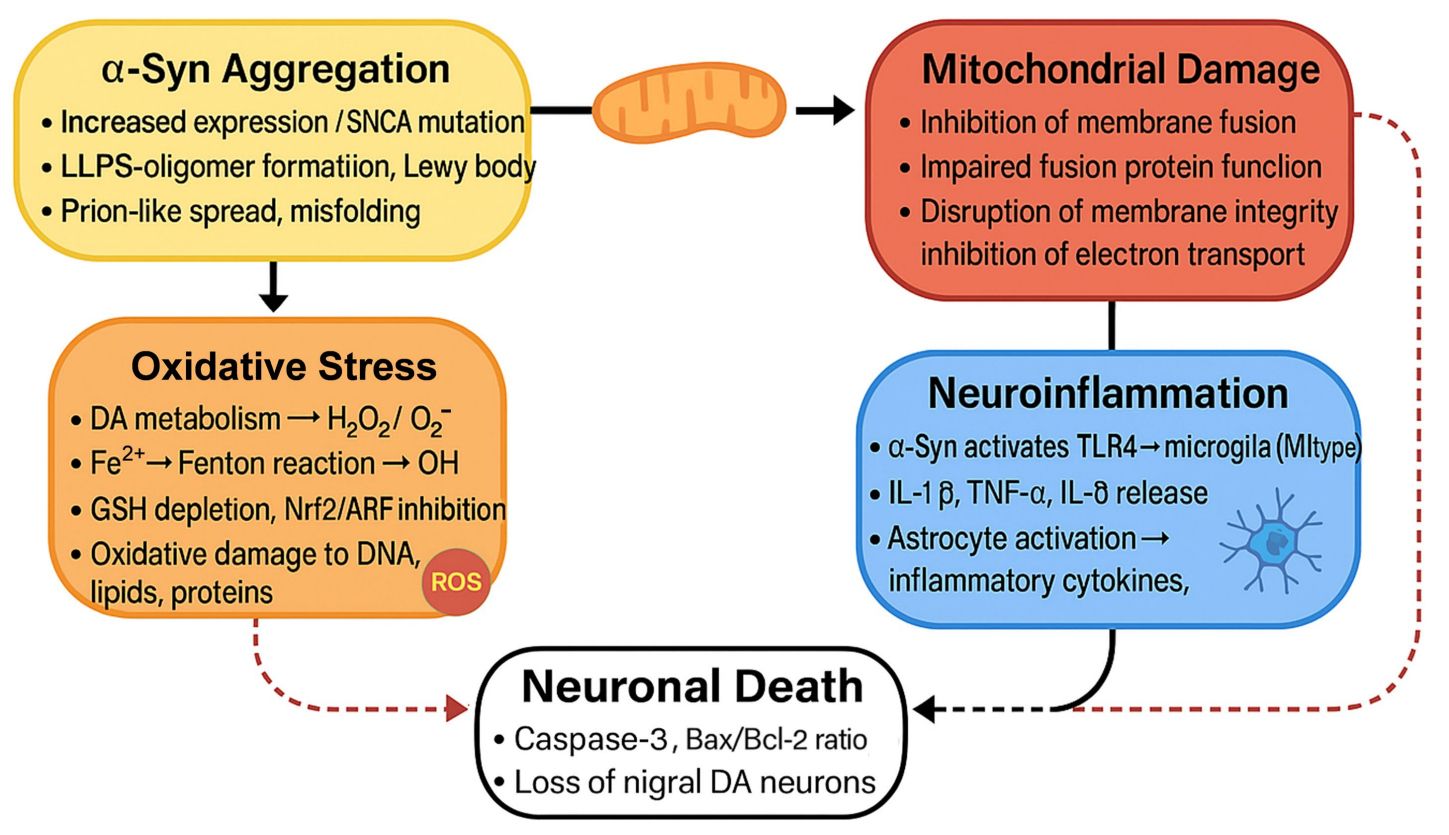
The Feedback Loop of α-syn Aggregation and Neuronal Death. This figure illustrates a chain reaction in which the aggregation of α-syn causes mitochondrial damage, oxidative stress, and neuroinflammation, ultimately leading to neuronal death. The process encompasses the activation of microglia and astrocytes, oxidative damage to cellular components, and disruption of cellular functions, thereby promoting the progression of PD.

Dopaminergic neurons, which are highly dependent on mitochondrial oxidative phosphorylation for energy, are particularly susceptible to α-syn-mediated mitochondrial damage. In the LB of patients with PD, α-syn, lipids, lysosomal components, and mitochondrial fragments can be detected (Shahmoradian et al., 2019). This finding indicates that *α-syn* aggregation, mitochondrial dysfunction, and damage to the lysosomal degradation system form a highly interconnected pathological network (Figure 1).

#### 2.3.2 Oxidative stress amplification

Oxidative stress plays a pivotal role in the pathogenesis of PD. The brain, accounting for only 2% of the body’s weight, yet consuming approximately 20% of the oxygen, making it particularly susceptible to oxidative stress (Michailidis et al., 2022). Oxidative stress refers to an imbalance between the generation and clearance of ROS, resulting in cellular damage.

During dopamine (DA) metabolism, highly ROS are produced, placing dopaminergic neurons in a higher oxidative state compared to other neuronal populations (Sadaka et al., 2023). These ROS are primarily generated through the enzymatic activity of monoamine oxidase (MAO) and other enzymes involved in DA metabolism. Under conditions of oxidative stress, DA can be metabolized through various pathways, including those mediated by MAO, leading to the production of significant amounts of hydrogen peroxide (H_2_O_2_) and superoxide anion (O^2−^).

Furthermore, the role of Fe^2+^ in ROS metabolism further exacerbates oxidative damage in the substantia nigra. Under normal conditions, iron is primarily stored in ferritin as ferric iron (Fe^3+^), which plays a crucial role in maintaining cellular iron homeostasis (Cadenas et al., 2019). Research has demonstrated that the levels of ferrous iron (Fe^2+^) are abnormally elevated in the substantia nigra of patients with PD (Wu et al., 2021). This accumulation of Fe^2+^ is closely associated with the heightened expression of divalent metal transporter 1 (DMT1) (Jiang et al., 2003; Guo et al., 2014; Moos and Morgan, 2004). The expression of DMT1 is significantly increased in the substantia nigra of PD patients, resulting in enhanced cellular uptake of Fe^2+^. Furthermore, Fe^2+^ can catalyze the generation of more toxic hydroxyl radicals (·OH) from H_2_O_2_ through the Fenton reaction (Oracz and Zyzelewicz, 2019), thereby exacerbating oxidative stress levels.

Under normal physiological conditions, antioxidants in the human body can effectively eliminate excess free radicals generated by the oxygen metabolism of brain tissue and repair cells damaged by oxidative damage. However, in patients with PD, factors such as glutathione (GSH) depletion, impaired function of the Nrf2/ARE signaling pathway, and mitochondrial dysfunction contribute to a significant decrease in antioxidant levels in the substantia nigra region. This reduction leads to the accumulation of free radicals and exacerbates oxidative damage. These free radicals are highly reactive toward polyunsaturated fatty acids, which are abundant in brain cellular membranes. Consequently, the elevated levels of free radicals can damage cellular structures, resulting in the accumulation of cytotoxic substances and ultimately leading to the death of substantia nigra cells, thereby triggering the onset of PD (Chittasupho et al., 2022).

#### 2.3.3 Neurological inflammation loop

Neuroinflammation is a significant indicator of PD and plays a pivotal role in its pathogenesis and progression (Kubota et al., 2020). As a key pathological feature, neuroinflammation refers to the immune response of microglia and astrocytes in the central nervous system to injury or pathogens (Chen et al., 2024). Under normal physiological conditions, microglia and astrocytes protect neurons by eliminating pathogens and cellular debris, as well as releasing neurotrophic factors, thereby maintaining homeostasis. However, the onset of PD disrupts this balance.

Studies have demonstrated that the abnormal aggregation of α-syn is a significant trigger of neuroinflammation in PD (Park et al., 2021). This abnormal aggregation can activate microglia via pattern recognition receptors (PRRs), including Toll-like receptor 4 (TLR4), thereby initiating an inflammatory response. The overactivation of microglia leads to the release of pro-inflammatory factors such as Interleukin-1 beta (IL-1β), Tumor Necrosis Factor-alpha (TNF-*α*), and Interleukin-6 (IL-6), which directly damage dopaminergic neurons in the substantia nigra pars compacta (SNpc) (McCarty and Lerner, 2020).

This phenomenon has been corroborated by autopsy findings in PD patients, which reveal a significant presence of activated microglia in the substantia nigra and basal ganglia regions of their brains, whereas microglia in the brain tissue of healthy individuals remain in a quiescent state (He et al., 2018). Moreover, neuroinflammation can exacerbate α-syn aggregation through intracellular signaling pathways, creating a vicious cycle. Inflammatory responses associated with PD induce the activation of the Nuclear Factor kappa-light-chain-enhancer of activated B cells (NF-κB) signaling pathway, which promotes the overexpression of α-syn. In turn, abnormal α-syn can further intensify inflammatory responses, thereby accelerating the progression of PD. Studies have demonstrated that in 1-Methyl-4-phenyl-1,2,3,6-tetrahydropyridine (MPTP) and 6-Hydroxydopamine (6-OHDA)-induced PD animal models, the activation of microglia and the release of pro-inflammatory factors are key mechanisms underlying dopaminergic neuronal death. When MPTP damages nigral dopaminergic neurons, the cellular debris and inflammatory mediators released by these neurons can further activate microglia, leading to a substantial release of inflammatory factors and the formation of a self-reinforcing vicious cycle. Similarly, in the 6-OHDA-treated rat PD model, an increase in microglia, activation of astrocytes, loss of dopaminergic neurons, and significant upregulation of pro-inflammatory factors (IL-1β, IL-6, TNF-*α*, IFN-*γ*) were observed, alongside a decrease in anti-inflammatory factor levels.

Furthermore, astrocytes are also integral to the process of neuroinflammation. Research indicates that astrocyte activation is closely linked to the progression of neurodegenerative diseases, particularly in PD, where it correlates with the degeneration of dopaminergic neurons (Kwon and Koh, 2020). The activation of astrocytes triggers the release of pro-inflammatory cytokines, such as TNF-*α* and IL-1β, which further intensify neuroinflammation and neurodegeneration (Chen et al., 2023). Inhibiting astrocyte activation or their pro-inflammatory signaling pathways, such as IKK2/NF-κB, can effectively mitigate neuroinflammation and protect neuronal health (Kirkley et al., 2019). These findings offer novel strategies for treating PD and provide theoretical support for the anti-inflammatory effects of molecular hydrogen.

## 3 Available treatments for PD

Currently, PD remains incurable, with treatment primarily focused on symptom management. Commonly employed treatment methods include drug therapy, surgical interventions, and adjunct therapies such as exercise and psychotherapy. These multidisciplinary approaches are often used in combination to address the complex motor and non-motor symptoms of PD.

### 3.1 Pharmacological treatment

Drug therapy is the most prevalent and widely utilized treatment for PD, primarily categorized into three groups: dopaminergic symptom treatment drugs, non-dopaminergic symptom treatment drugs, and disease-modifying treatment drugs. Medications for the treatment of dopaminergic symptoms include encompassing DA replacement therapy, peripheral 3,4-Dihydroxyphenylalanine (DOPA) decarboxylase inhibitors (DCI), DA receptor (DR) agonists, MAO-B inhibitors, and catechol-O-methyltransferase (COMT) inhibitors.

Levodopa is regarded as the ‘gold standard’ for PD treatment, effectively alleviating motor symptoms (Calabresi and Standaert, 2019). Additionally, it serves as the cornerstone of DA replacement therapy. This dopaminergic replacement therapy is converted into DA across the blood–brain barrier, leading to rapid improvements in bradykinesia and rigidity. However, prolonged use of levodopa may result in diminished drug efficacy and the emergence of motor complications (Poewe et al., 2017), including the ‘on–off’ phenomenon and dyskinesia. To enhance the efficacy of levodopa and reduce peripheral adverse effects, it is commonly combined with a decarboxylase inhibitor in clinical practice (van Rumund et al., 2021).

DCI do not directly improve the symptoms of PD. Instead, they function by inhibiting the activity of peripheral DOPA decarboxylase (DDC), which reduces the conversion of levodopa in peripheral tissues and increases its availability in the central nervous system, while also minimizing side effects such as nausea and vomiting (Gremke et al., 2022). Consequently, decarboxylase inhibitors are a core component of DA replacement therapy. Clinically, the decarboxylase inhibitors carbidopa and benserazide are frequently formulated into combination preparations with levodopa, representing a standard combination therapy for PD (van Rumund et al., 2021).

In addition to levodopa, DA receptor agonists—such as pramipexole, ropinirole, and rotigotine—can also be utilized in the treatment of PD, particularly for early-stage patients or in conjunction with levodopa to delay the onset of motor complications (Herz et al., 2015). By directly activating DA receptors, these medications can improve motor symptoms; however, long-term use may lead to non-motor symptoms, including drowsiness and hallucinations. New DA receptor agonists, such as tavapadon, are currently under investigation and may reduce the risk of motor complications.

In addition to directly supplementing or activating DA receptors, prolonging the duration of endogenous DA action represents another important strategy. MAO-B inhibitors enhance DA levels in the central nervous system by inhibiting DA degradation, thereby alleviating motor symptoms of PD symptoms (Fox et al., 2018). Typically, MAO-B inhibitors are used in combination with levodopa to enhance efficacy and reduce motor symptom fluctuations. However, long-term use may increase the risk of adverse effects, particularly non-motor symptoms such as hallucinations and sleep disturbances.

Similar to MAO-B inhibitors, COMT inhibitors, such as entacapone and tolcapone, can extend the duration of levodopa’s action in the central nervous system, thereby enhancing therapeutic efficacy. However, the efficacy of COMT inhibitors is limited when used alone; thus, they are typically combined with levodopa to improve treatment outcomes and alleviate ‘off’ symptoms (Rangasamy et al., 2019). Prolonged use may lead to dyskinesia, and tolcapone poses a risk of hepatotoxicity, necessitating regular monitoring of liver function.

In addition to dopaminergic drugs, non-dopaminergic medications also play a significant role in the treatment of PD. These drugs are primarily utilized to improve non-motor symptoms, particularly when managing motor complications proves challenging. Amantadine, an NMDA receptor antagonist, has been shown to reduce levodopa-induced dyskinesia (Jansen et al., 2014). Pimavanserin, a 5-HT2A receptor inverse agonist, effectively alleviates PD-related hallucinations and delusions without exacerbating motor symptoms (Bugarski-Kirola et al., 2022). Adenosine A2A receptor antagonists, such as istradefylline, can relieve ‘off’ periods and enhance patients’ motor functions (Rascol et al., 2015). However, due to the potential for these medications to induce cognitive decline, especially in elderly patients, their usage has become less common in recent years. Overall, non-dopaminergic medications provide additional options for PD treatment, particularly in managing motor complications and non-motor symptoms. While some drugs have received approval for clinical use, many new therapies are still in the trial phase and necessitate further research to confirm their efficacy and safety.

In recent years, disease-modifying therapy (DMT) has increasingly emerged as a focal point in PD research. Unlike traditional symptomatic treatments, the primary objective of DMT is to decelerate disease progression by intervening in the pathological mechanisms underlying PD (Elkouzi et al., 2019; Wolff et al., 2023). Current research avenues encompass α-syn-targeted therapies, GBA-targeted therapies, GLP-1 receptor agonists, and antioxidative stress medications. Although these therapeutic approaches have demonstrated promise in preclinical studies, no DMT drugs have yet received approval, and many candidate drugs have failed to achieve primary endpoints in clinical trials. Consequently, further research is essential to elucidate their efficacy and safety, as well as to advance the development of personalized treatment strategies. The currently approved pharmacological treatments for PD are summarized in Table 1.

**Table 1:** PD treatment medications.

Drug class	Approved drugs (generic/brand)	Mechanism of action	Daily dose range	Common side effects (≥5%)
DA precursors	Carbidopa/Levodopa (Sinemet^®^)	Levodopa converts to DA in the brain; carbidopa inhibits peripheral metabolism	300–1,000 mg (divided into 3–4 doses)	Motor complications (dyskinesia, wearing-off), nausea, orthostatic hypotension
DA receptor agonists	Pramipexole (Mirapex^®^), Rotigotine (Neupro^®^)	Activates D2/D3 receptors to mimic DA signaling	Pramipexole: 0.375–4.5 mg; Rotigotine: 2–8 mg/24 h (patch)	Somnolence, impulse control disorders, peripheral edema
MAO-B inhibitors	Rasagiline (Azilect^®^), Safinamide (Xadago^®^)	Inhibits DA-degrading enzyme MAO-B to prolong DA activity	Rasagiline: 0.5–1 mg/day; Safinamide: 50–100 mg	Headache, insomnia, arthralgia (co-administration with SSRIs may increase risk of serotonin syndrome)
COMT inhibitors	Entacapone (Comtan^®^), Opicapone (Ongentys^®^)	Inhibits peripheral levodopa metabolism to enhance efficacy	Entacapone: 200 mg (with each levodopa dose); Opicapone: 50 mg once daily	Diarrhea, orange-red urine, increased liver enzymes
Continuous infusion	Carbidopa/Levodopa (Duopa^®^)	24-h intestinal infusion for stable plasma concentration	Levodopa 60 mg/mL + Carbidopa 7.5 mg/mL (individualized)	Infusion site reactions (erythema, nodules, infection)
Anticholinergics	Trihexyphenidyl (Artane^®^)	Reduces acetylcholine activity to correct neurotransmitter imbalance	1–6 mg (divided into 2–3 doses)	Cognitive decline, dry mouth, urinary retention (contraindicated in elderly)
NMDA antagonists	Amantadine (Symmetrel^®^)	Promotes DA release and modulates glutamatergic pathways	100–400 mg (divided into 2 doses; last dose before 4 PM)	Livedo reticularis, hallucinations, insomnia
Adenosine A2A antagonists	Istradefylline (Nourianz^®^)	Blocks adenosine receptors to enhance DA signaling	20–40 mg once daily	not approved in China (approved in US, Japan)

### 3.2 Surgical treatment

For patients exhibiting a poor response to drug therapy or experiencing severe complications, surgical intervention may be considered as an alternative treatment option. One such procedure is Pallidotomy, which targets the ventrointermediate nucleus of the thalamus (Vim) and the posterior part of the globus pallidus for therapeutic ultrasound treatment, while carefully monitoring for adverse neurological reactions such as postoperative movement disorders or cognitive dysfunction. However, due to the significant tissue damage and high recurrence rates associated with this surgery, it has become largely obsolete (Bond et al., 2017). Another surgical option is deep brain stimulation (DBS), which involves the implantation of electrodes in the subthalamic nucleus (STN) or the medial part of the globus pallidus (GPi), offering a more targeted and reversible approach to symptom management. DBS can alleviate motor symptoms, reduce the required dosage of medication, and is considered less invasive and safer compared to neurodestructive surgery, making it the preferred method for surgical treatment at present (Hacker et al., 2018). Nonetheless, the surgical indications for DBS are relatively stringent, applying only to patients with a disease duration exceeding 5 years who demonstrate a good response to levodopa, and there are specific limitations (Fenoy and Simpson, 2014). These limitations include the risk of postoperative infection and complications related to the hardware, as well as a lack of significant improvement in axial symptoms such as freezing gait and postural imbalance. Furthermore, DBS does not halt the progressive loss of dopaminergic neurons in the substantia nigra, which limits its ability to slow disease progression.

### 3.3 Adjunctive therapy

Among various adjunctive treatment methods, exercise therapy—including Tai Chi and resistance training—has been shown to enhance balance function in the short term, though its long-term benefits remain uncertain (Shen et al., 2016). Additionally, psychological interventions, such as cognitive-behavioral therapy (CBT), can alleviate depressive symptoms, evidenced by a reduction in the Hamilton Depression Rating Scale (HAMD) score by 3 to 5 points (Dobkin et al., 2011). However, it is important to note that these methods currently lack robust evidence supporting their long-term efficacy, particularly in terms of sustained symptom improvement and quality of life enhancement.

Although existing drug and surgical treatments can alleviate the motor symptoms of PD to some extent, they remain inadequate in delaying the progression of the disease and improving non-motor symptoms. This underscores the importance of researching new adjunctive treatment methods.

In recent years, an increasing number of studies have focused on adjunctive therapeutic strategies aimed at further protecting dopaminergic neurons based on existing treatments, delaying disease progression, and improving patients’ quality of life. In this context, molecular hydrogen (H₂) has emerged as a potential adjunctive treatment for PD due to its excellent biosafety and neuroprotective effects. Given the complex multi-level pathological characteristics of PD, particularly the neurodegenerative changes associated with α-syn aggregation, molecular hydrogen, through its selective antioxidant and anti-inflammatory properties, demonstrates multi-target regulatory effects, offering new possibilities for slowing the disease course.

## 4 Mechanism of neuroprotective action of hydrogen

As the smallest molecule with exceptional tissue penetrability, H_2_ exhibits unique antioxidant and anti-inflammatory properties, enabling it to effectively combat oxidative stress and inflammation. These characteristics highlight its potential role not only as a neuroprotective agent but also as an adjunctive therapy in comprehensive treatment strategies for PD. It was traditionally believed that H_2_ is an inert gas until 1975, when Dole et al. (1975) demonstrated that high-pressure hydrogen could effectively inhibit the growth of squamous cell carcinoma in mice. Fukuda et al. (2007) from Japan first discovered that hydrogen can selectively scavenge OH and peroxynitrite, which are key contributors to oxidative stress and cellular damage. Since that time, numerous studies have demonstrated that H_2_ has significant therapeutic effects on various diseases, including those related to brain oxidative stress, liver and intestinal transplantation, myocardial injury, and atherosclerosis (Fukuda et al., 2007; Nagata et al., 2009; Gu et al., 2010; Buchholz et al., 2008; Ohsawa et al., 2008). The therapeutic effects of hydrogen on these conditions are closely linked to its antioxidant, anti-inflammatory, and anti-apoptotic properties, which collectively contribute to its therapeutic potential. In addition, recent studies have further confirmed that hydrogen therapy can modulate oxidative stress, neuroinflammation, and gut-brain axis dysregulation in neurodegenerative disorders such as Alzheimer’s disease (He et al., 2023; He et al., 2024). These findings provide a strong rationale for exploring hydrogen’s potential neuroprotective effects in PD as well.

### 4.1 Antioxidant mechanism of hydrogen

Hydrogen exhibits a favorable neuroprotective mechanism in neurodegenerative diseases, with its effects potentially involving antioxidant, anti-inflammatory, and anti-apoptotic pathways. Numerous neurological disorders are closely associated with oxidative stress, which is often implicated in the pathogenesis of neurodegenerative diseases (Novosadova et al., 2022). As an antioxidant, H_2_’s unique advantage lies in its ability to selectively scavenge toxic free radicals. It effectively neutralizes highly reactive radicals, such as ·OH and ONOO^−^, while sparing normally physiologically active free radicals like O^2−^, nitric oxide (NO), and H_2_O_2_. This selectivity enables hydrogen to mitigate oxidative damage without disrupting the body’s endogenous homeostasis. It is noteworthy that the longer half-life of ONOO^−^ compared to OH may increase the likelihood of its reaction with hydrogen gas. Previous studies have demonstrated that hydrogen gas can inhibit the formation of nitrotyrosine (Zhang et al., 2016), a detectable marker for the indirect measurement of ONOO^−^ (Wu et al., 2018), suggesting that hydrogen gas may exert its antioxidant effects by inhibiting ONOO^−^. In the study Fukuda et al. (2007), it was proposed that H₂ might exert antioxidant effects by directly scavenging ONOO^−^/ONOOH. However, in Penders’ study, it was shown through stopped-flow spectroscopy and ion chromatography analysis that the addition of H₂ did not alter the decomposition rate of ONOOH and did not significantly increase the proportion of NO₂^−^ in the decomposition products of ONOOH. Stopped-flow experiments mixing ONOOH with tyrosine derivatives demonstrated that H₂ does not affect the nitration efficiency of ONOOH on tyrosine. Therefore, Penders explicitly rejected the hypothesis that H₂ directly reacts with ONOOH or inhibits tyrosine nitration, clarified related controversies, and pointed out that the ‘antioxidant’ effect of H₂ is more likely achieved through other mechanisms (such as signal regulation) rather than through direct scavenging of reactive oxygen/nitrogen species (Penders et al., 2014).

In the neuroprotective mechanism of H_2_, its antioxidant effect is manifested not only through the direct neutralization of ·OH but also via the inhibition of NADPH oxidase activity, thereby exerting antioxidant efficacy. NADPH oxidase is a pro-oxidant enzyme (Erlich et al., 2022) that facilitates the transfer of electrons from NADPH to oxygen, generating O2· and other downstream ROS (Bedard and Krause, 2007). Together with six homologs of the cytochrome subunit of NADPH oxidase—NOX1, NOX3, NOX4, NOX5, DUOX1, and DUOX2—it constitutes the NOX enzyme family. Increased NOX activity can lead to various pathologies, particularly cardiovascular diseases and neurodegenerative disorders (Bedard and Krause, 2007). In studies investigating the regulation of oxidative stress in mast cells by H_2_ (Itoh et al., 2009), it was found that H₂ reduces the levels of NADPH oxidase subunits (such as p40 phox, p47 phox, and p67 phox) in the cell membrane while increasing their levels in the cytoplasm, selectively interfering with the membrane localization of these subunits. By limiting the transport of these molecules to the cell membrane, H_2_ decreases NADPH oxidase activity, reduces the generation of ROS, and ultimately achieves an antioxidant effect.

The antioxidant effect of H_2_ is not solely dependent on the direct scavenging of ROS; it also enhances the endogenous defense system by modulating the Nrf2/ARE pathway. Nrf2 serves as a crucial defense mechanism in the brain against toxins in glial and neuronal cells (Katoh et al., 2001; Guo et al., 2019). In a normal or resting state, Nrf2 molecules are tightly bound in the cytoplasm by Keap-1, rendering them inactive. However, under oxidative stress, oxidative modification of cysteine residues in Keap1 causes conformational changes that weaken its binding to Nrf2, allowing Nrf2 to dissociate from Keap-1 and rapidly translocate to the nucleus. There, Nrf2 binds to antioxidant response elements (ARE) located in the promoter regions of target genes, initiating the transcription of cytoprotective genes and subsequently upregulating the expression of HO-1 and superoxide dismutase 1 (SOD1). This process enhances intracellular antioxidant expression and elevates the levels of intracellular antioxidant enzymes, thereby down-regulating oxidative stress (Yang et al., 2021; Ruan et al., 2019). Innamorato et al. (2010) demonstrated through knockout experiments that Nrf2-deficient mice exhibited heightened susceptibility to the MPTP-induced PD model, resulting in significantly lower survival rates of dopaminergic neurons. This indicates that Nrf2 plays a crucial role in PD by regulating antioxidant responses and alleviating neuroinflammation, thereby providing neuroprotection in PD. Studies have shown that H_2_ can activate Nrf2 and induce its translocation into the nucleus, enhancing the transcription of catalase (CAT) and glutathione peroxidase 1 (GPX1) (Murakami et al., 2017), thereby upregulating the expression of intracellular antioxidant enzymes through the Nrf2-ARE pathway, neutralizing ROS, mitigating oxidative damage, and ultimately achieving systemic antioxidant protection of hydrogen at the cellular and mitochondrial levels.

Furthermore, in comparison to conventional antioxidants, such as vitamin E, hydrogen demonstrates superior efficacy in penetrating core damage regions, including mitochondria, owing to its small molecular size and high diffusibility. A detailed comparison between hydrogen and traditional antioxidants such as vitamins E and C is provided in Table 2. Given the oxidative stress associated with long-term levodopa therapy, H₂ antioxidant function may provide additional benefits when used in combination, potentially mitigating treatment-related oxidative damage.

**Table 2:** Comparative analysis of H₂ and vitamin E/C as antioxidants.

Comparison item	H₂	VE	VC
Selectivity	selective	Non-selective	Non-selective
Antioxidant properties	Scavenges ⋅OH, preserves physiological ROS (Fukuda et al., 2007)	Inhibits lipid peroxidation (Niu et al., 2024; Man Anh et al., 2019)	Broad-spectrum ROS scavenger (Chang et al., 2021; Jakel et al., 2007)
Blood–brain barrier penetration	Small and hydrophobic molecules, easily penetrate the blood–brain barrier	lipophilic, passive diffusion, exhibiting better central nervous system permeability than VC (Sharma et al., 2016)	hydrophilic, requires active transport (Sharma et al., 2016)
Neuroprotective mechanism	Reduces oxidative stress, activates Nrf2/ARE (Fukuda et al., 2007)	Supports mitochondria, reduces inflammation (Niu et al., 2024)	maintains mitochondrial membrane integrity, alleviates neuronal degeneration (Chang et al., 2021; Jakel et al., 2007)
Side effects and safety	No significant adverse effects	High doses of VE (>400 IU/day) may increase bleeding risk (Sharma et al., 2016)	Excessive intake may cause GI discomfort

### 4.2 Anti-inflammatory mechanisms of hydrogen

Numerous studies have demonstrated that hydrogen possesses significant anti-inflammatory properties across a range of diseases, including metabolic syndrome, ischemia–reperfusion injury, inflammatory bowel disease, cancer, and alcoholic liver disease (Hu et al., 2024; Yang et al., 2018; Lin et al., 2017). The anti-inflammatory effects of H₂ are mediated through multiple mechanisms. H₂ significantly reduces the release of pro-inflammatory factors, including IL-1*β*, IL-6, TNF-*α*, NF-κB, and HMGB1, while simultaneously increasing the levels of anti-inflammatory factors such as IL-4, IL-10, and IL-13. Furthermore, H₂ promotes the polarization of macrophages from the pro-inflammatory M1 phenotype to the anti-inflammatory M2 phenotype, thereby inducing the secretion of anti-inflammatory mediators like IL-10 and TGF-β, which contributes to the establishment of a positive regulatory feedback loop. Certainly, H₂ can also exert anti-inflammatory effects through multiple inflammatory signaling pathways. The NF-κB pathway, a central hub of inflammation, is inhibited by H₂ in various diseases, thereby blocking downstream inflammatory cascades (Sim et al., 2020). In a study utilizing a neonatal mouse model of hypoxic–ischemic brain injury, it was demonstrated that hydrogen activates the Nrf2 pathway to enhance intracellular antioxidant defenses, effectively clearing excess ROS. This process inhibits ROS-mediated IKK kinase phosphorylation, blocks IκB*α* protein degradation, and prevents the nuclear translocation of the NF-κB p65 subunit. Further experiments revealed that hydrogen-induced Nrf2 can directly bind to NF-κB p65, interfering with its DNA-binding ability to pro-inflammatory gene promoters while also suppressing the release of inflammatory mediators such as HMGB1 and TNF-α, thereby truncating the positive feedback loop of IKK. Through these triple mechanisms of Nrf2-dependent redox regulation, protein interaction, and inflammatory signaling blockade, hydrogen achieves synergistic inhibition of the NF-κB signaling pathway (Hu et al., 2022). Additionally, the NLRP3 inflammasome pathway mediates both acute and chronic inflammation, and H₂ demonstrates a specific inhibitory effect on its activation (Meyers and Zhu, 2020; Yang et al., 2020). Recent studies on Alzheimer’s disease models have demonstrated that hydrogen can mitigate neuroinflammation by inhibiting the NLRP3 inflammasome pathway, thereby reducing the expression of IL-1β and TNF-*α*, and modulating microglial polarization. This mechanism contributes to the attenuation of neuronal damage (He et al., 2023). It is noteworthy that the anti-inflammatory effects of H₂ are especially pronounced in the nervous system (Nethathe et al., 2024).

In the pathological progression of PD, the inflammatory response initiated by aberrant microglial activation plays a crucial role in the degeneration of dopaminergic neurons. In PD, persistent microglial activation and the production of ROS lead to the release of pro-inflammatory cytokines, establishing a self-perpetuating cycle (Badanjak et al., 2021; Isik et al., 2023).

M1/M2 polarization of microglia plays a crucial role in neuroinflammation (Qin et al., 2019). It has been reported that promoting the conversion of microglia to the M2 phenotype contributes to a reduction in neuroinflammation in PD (Yang et al., 2021; Li et al., 2022; Calvello et al., 2017). In a model of sepsis-induced neuroinflammation, hydrogen has been shown to decrease the polarization of M1-type microglia while increasing the polarization of M2-type microglia through the modulation of mTOR-autophagy-dependent pathways (Zhuang et al., 2020). Researchers have discovered that hydrogen intervention in sepsis models, specifically CLP mice and LPS-induced BV-2 cells, significantly inhibits the phosphorylation level of mTOR (indicated by a decrease in the p-mTOR/mTOR ratio) while activating the AMPK signaling pathway (indicated by an increase in the p-AMPK/AMPK ratio). As a cellular energy sensor, the activation of AMPK further suppresses mTOR activity. Conversely, mTOR, which acts as a negative regulator of autophagy, exhibits reduced activity, leading to the dephosphorylation of the downstream autophagy initiation protein ULK1. This reduction lifts the inhibition on autophagy and triggers autophagosome formation, as evidenced by increased levels of LC3-II/I and Beclin-1, alongside a decrease in p62. This process alleviates sepsis-associated neuroinflammation, as indicated by elevated hippocampal IL-10 levels, and cognitive impairment, demonstrated by reduced water maze escape latency, through the clearance of pro-inflammatory factors such as TNF-*α* and IL-6, which are also decreased. Furthermore, it promotes the polarization of microglia from the pro-inflammatory M1 phenotype (indicated by a decrease in the CD86 + ratio) to the anti-inflammatory M2 phenotype (indicated by an increase in the CD206 + ratio). Mechanistic validation revealed that the use of the mTOR activator MHY1485 reversed the anti-inflammatory effects of hydrogen, thereby confirming its regulation of microglial transformation via the mTOR-autophagy axis and providing molecular evidence for hydrogen therapy in sepsis-associated encephalopathy (SAE) (Zhuang et al., 2020). Similarly, in an ischemic stroke model, hydrogen significantly inhibited the increase in M1-type microglia (Ning et al., 2018).

### 4.3 Anti-apoptotic mechanisms of hydrogen

In addition to its antioxidant and anti-inflammatory properties, the hydrogen molecule exhibits significant anti-apoptotic effects, which have been extensively studied across various neurological disorders. For instance, in animal models of subarachnoid hemorrhage, hypoxic–ischemic brain injury, and vascular dementia, hydrogen markedly reduced the onset of apoptosis, thereby providing robust neuroprotective benefits (Hong et al., 2014; Wang et al., 2020; Jiang et al., 2019; Lee and Choi, 2021). Furthermore, hydrogen-rich water has been demonstrated to facilitate the repair of peripheral nerve injuries through its anti-apoptotic effects. Notably, low-dose hydrogen water treatment has been shown to enhance axonal regeneration and recovery of neurological function, suggesting its potential for clinical translation (Zhang et al., 2015).

In PD, apoptosis plays a significant role in the pathological mechanisms underlying the loss of dopaminergic neurons in the SNpc caused by oxidative stress and mitochondrial dysfunction. Pathological studies have confirmed that the expression of the pro-apoptotic protein Bax is significantly upregulated in Lewy body-positive dopaminergic neurons of patients with PD (Bové et al., 2014). Moreover, an elevated Bax/Bcl-2 ratio is considered a critical factor in the degenerative pathology of dopaminergic neurons. One study indicated that down-regulating the expression of the pro-apoptotic factor Bax and reducing the Bax/Bcl-2 ratio effectively mitigated degenerative lesions in dopaminergic neurons associated with PD (Yang et al., 2025). These findings suggest that hydrogen alleviates the effects of PD in part by modulating Bax expression and the Bax/Bcl-2 ratio, which are key mechanisms underlying its anti-apoptotic action. Research on Alzheimer’s disease models has demonstrated that hydrogen therapy can reduce cell apoptosis by modulating key apoptotic regulators such as Bax and caspase-3, thereby promoting neuronal survival (He et al., 2023).

**Figure 2:**
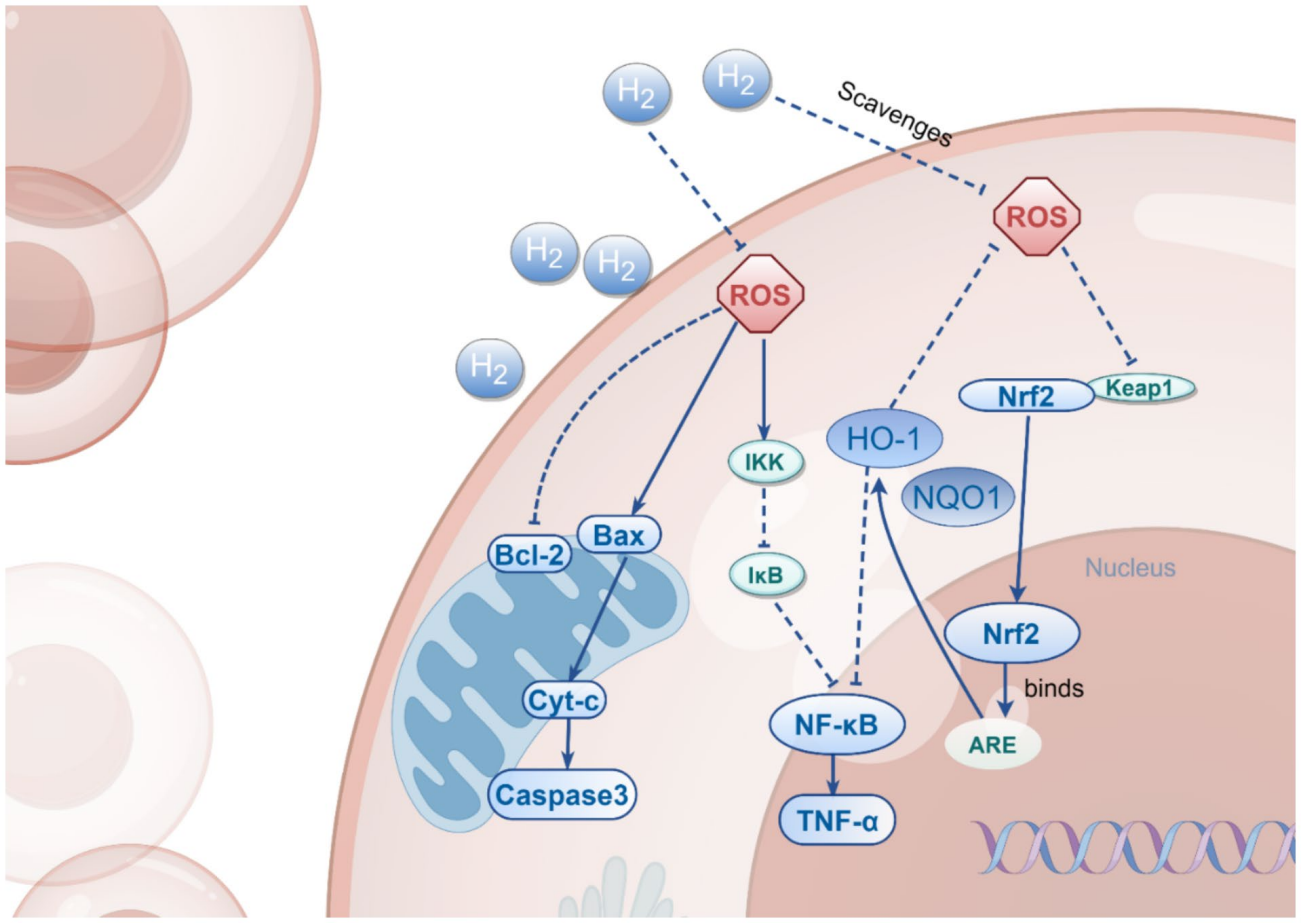
The Multi-Target Effects of H_2_. This figure illustrates the multiple mechanisms by which H₂ operates in cells, including the activation of Nrf2, inhibition of NF-κB, and regulation of Bax/Bcl-2. H₂ exerts its cytoprotective effects through direct scavenging of ROS, inhibition of NF-κB pathway activation, and modulation of the expression of both anti-apoptotic and pro-apoptotic proteins, thereby influencing the processes of cell survival and death.

In a neonatal rat hypoxic–ischemic brain injury (HIBI) model, hydrogen significantly reduced the Bax/Bcl-2 ratio by up-regulating the expression of the anti-apoptotic protein Bcl-2, while down-regulating the expression of the pro-apoptotic protein Bax and the apoptosis-executing enzyme caspase-3, leading to a reduction in apoptosis (Wang et al., 2020). Additionally, hydrogen has demonstrated a similar anti-apoptotic mechanism in other neurological diseases (Hong et al., 2014; Wang et al., 2020; Jiang et al., 2019; Zhuang et al., 2013; Li et al., 2018), where it exerts its neuroprotective effects primarily by inhibiting the expression of pro-apoptotic factors such as Bax, caspase-3, and caspase-12, while promoting the expression of anti-apoptotic factors Bcl-2 and Bcl-xL. Collectively, the anti-apoptotic mechanism of hydrogen is both universal and consistent across various neurological conditions, underscoring its potential as a novel therapeutic agent. By modulating the expression of apoptosis-related proteins, hydrogen effectively reduces apoptosis, thereby exerting neuroprotective effects. Given its demonstrated efficacy in reducing apoptosis in other neurological diseases, this mechanism holds significant application potential in PD, providing a theoretical foundation for hydrogen as a novel neuroprotective agent in the treatment of PD. These multiple mechanisms of action, including antioxidant, anti-inflammatory, and anti-apoptotic pathways, are integrated and visually summarized in Figure 2. To facilitate a more intuitive comparison between hydrogen therapy and traditional treatments, such as Levodopa and deep brain stimulation, we have created Table 3, which summarizes the main differences in terms of brain penetrability, safety, and clinical applicability.

**Table 3:** Hydrogen vs. conventional treatments.

Aspect	Hydrogen therapy	Traditional treatments (e.g., Levodopa, DBS)
Brain Penetration	Small molecule gas, effectively crosses the blood–brain barrier, widely distributed in the nervous system.	Traditional treatments like levodopa and deep brain stimulation are effective but have limited distribution in the nervous system.
Safety	Safe at low concentrations with no significant adverse effects observed. Higher safety profile for long-term use.	Levodopa and DBS are safe, but long-term levodopa use may lead to diminishing efficacy, and DBS carries surgical risks.
Clinical Applicability	Promising therapeutic effects in early trials, but large-scale studies are needed for wider applicability.	While traditional treatments show efficacy, they face issues like diminishing effects over time and limited scope, especially in advanced stages.

## 5 Administration routes of H_2_

Hydrogen can be administered or ingested into the body through various routes, which can be primarily categorized into three types: gas inhalation, drinking hydrogen-rich water, and injecting hydrogen-rich saline. Each method has distinct advantages and limitations.

### 5.1 H_2_ inhalation

H_2_ inhalation is the simplest and most commonly used method, as H_2_ can easily diffuse through the alveoli and be delivered throughout the body, ensuring widespread systemic effects. From a toxicity perspective, hydrogen demonstrates a significant advantage over other medical gases. Even at high concentrations, hydrogen remains non-toxic and harmless, and it has been widely utilized in the field of diving (Qian et al., 2020). Research indicates that inhaling hydrogen does not affect blood pressure or other blood parameters such as pH and body temperature, and no significant adverse effects have been observed (Wang et al., 2021).

H_2_ inhalation has demonstrated a neuroprotective effect. Peng et al. (2024) found that inhaling 3% H_2_ can exert a neuroprotective effect by inhibiting neuronal iron death and reducing neuroinflammation following spontaneous subarachnoid hemorrhage (SAH). However, H_2_ concentrations in the air exceeding 4% pose an explosion risk. Consequently, the concentration of the H_2_ mixed gas is typically maintained between 1 and 4%.

In terms of hydrogen delivery methods, hydrogen can be connected to a ventilator circuit, mask, or nasal cannula, through which it is delivered to the lungs. These delivery methods facilitate the rapid diffusion of hydrogen throughout the body, offering protection against acute oxidative stress without influencing blood pressure. Liu et al. (2014) measured the hydrogen concentration in the tissues of rats that ingested hydrogen through different methods and found that oral and intraperitoneal injections can achieve higher hydrogen concentrations in multiple organs, while hydrogen inhalation results in elevated concentrations in muscle and brain tissues. These results strongly suggest that the inhalation route is the preferred method of administering H₂ for the treatment of central nervous system disorders. A comparative overview of the current hydrogen administration routes, along with their respective advantages and limitations, is provided in Table 4.

**Table 4:** Current administration methods of hydrogen and their advantages and disadvantages.

Administration routes for H_2_	Advantages	Disadvantages
Inhalation of hydrogen	Rapid action; high safety.	Concentration >4% poses an explosion risk
Drinking HRW	Safe, practical, convenient.	The solubility of hydrogen is low, requiring simultaneous intake of a large amount of liquid.
Intravenous or intraperitoneal injection of HRW	Achieves efficient and precise dosage delivery of H_2_	Requires professional medical procedures.

### 5.2 HRW

Drinking hydrogen-rich water is considered safer and more comfortable than hydrogen inhalation, as it avoids the risks associated with high-pressure gas exposure.

In recent years, the therapeutic potential of hydrogen-rich water (HRW) has garnered significant attention, particularly in the field of neurodegenerative diseases. With advancements in HRW preparation methods, such as water electrolysis (Santos et al., 2017), metal-acid reactions (Xie et al., 2020), and high-pressure dissolution, an increasing number of clinical trials have begun to adopt the consumption of hydrogen-rich water as a means of hydrogen intake for the treatment of neurological disorders. Yang et al. (2016) investigated the effects of hydrogen-rich water on neonatal hypoxic–ischemic encephalopathy, revealing that it is safe and exhibits certain therapeutic effects when neonates consume hydrogen-rich water (5 mL/kg) for 10 consecutive days, starting from 2 days after birth, in addition to conventional treatment. Furthermore, hydrogen-rich water (HRW) has also been utilized in research on the treatment of depression (Mizuno et al., 2017). Patients consumed 600 mL of HRW daily for 4 weeks, and the results indicated that HRW could enhance the function of the central nervous system by improving mood, alleviating anxiety, and regulating autonomic nervous function, thereby improving the quality of life.

Compared to direct inhalation of hydrogen, drinking HRW is more convenient and can be stored long-term in aluminum cans, which contributes to relatively higher patient compliance. However, due to the extremely low solubility of hydrogen molecules in water (1.6 ppm), a substantial volume of liquid must be ingested simultaneously when consuming oral hydrogen-rich saline.

In addition to being drinkable, HRW can also be administered via intravenous or intraperitoneal injection, allowing for precise control of hydrogen delivery and ensuring effective tissue penetration. Researchers investigated the therapeutic effects of hydrogen on acute ischemic stroke by administering intravenous hydrogen-rich saline. Patients received a combination of intravenous hydrogen-rich saline and Edaravone. After 7 days, clinical indicators and magnetic resonance imaging results demonstrated that the combined treatment significantly reduced the duration for patients to return to normal (Ono et al., 2011). Nagatani et al. (2013) also examined the effects of intravenous hydrogen-rich water and Edaravone on acute ischemic stroke. Their findings indicated that hydrogen-rich water is safe for the treatment of ischemic stroke, and its combination with existing treatment methods may provide additional benefits for patients. In a study focused on the treatment of severe subarachnoid hemorrhage, intravenous administration of hydrogen-rich liquid was also employed. This experiment involved the continuous combination of intravenous hydrogen-rich liquid with intrathecal injection of magnesium sulfate over a period of 14 days. The results revealed that the intrathecal infusion of magnesium sulfate, in conjunction with intravenous hydrogen therapy, could reduce serum malondialdehyde and NSE levels, as well as improve the Barthel index, further indicating that hydrogen has an adjunctive therapeutic effect (Takeuchi et al., 2021).

### 5.3 Nanocarriers

In traditional methods of hydrogen administration, the low solubility of H₂ presents a significant challenge, potentially hindering the ability to meet the required hydrogen concentrations for treating brain diseases. Therefore, it is crucial to explore methods that enhance hydrogen delivery to the brain. In this context, liposomes emerge as a promising nanocarrier, offering unique advantages.

Liposomes are nanoscale to microscale vesicles composed of one or more lipid bilayers that surround an aqueous compartment (Sanches et al., 2021). They exhibit excellent biocompatibility, biodegradability, and low immunogenicity, and are capable of encapsulating hydrophilic, lipophilic, and hydrophobic substances, making them ideal carriers for drug delivery (Gu et al., 2019). In the treatment of neurological disorders, the application of liposomes is particularly crucial, as the blood–brain barrier (BBB) restricts the entry of many drugs into the brain (Topal et al., 2020).

Liposomes enhance the efficiency of brain-targeted delivery through the synergistic action of multiple mechanisms. Their unique physicochemical properties enable the encapsulation of a variety of drugs, offering excellent biocompatibility and biodegradability. The modification of liposomes with active targeting ligands is of significant importance in enhancing brain-targeted delivery. Studies have shown that liposomes modified with the transferrin receptor antibody OX26 can activate receptor-mediated transcytosis, significantly improving the ability of drugs to cross the blood–brain barrier, thereby achieving sustained therapeutic effects in PD rat models (Xia et al., 2008). In the mechanism of electrostatic adsorption mediated by cationic charges, cationic lipids interact with the negative charges of the blood–brain barrier endothelial cells, facilitating drug delivery. Miglior’s study demonstrated that the intranasal administration of Glial cell line-derived neurotrophic factor (GDNF) plasmid to the brain regions of PD models resulted in neurotrophic and neuroprotective effects in PD rat models (Migliore et al., 2014). Additionally, PEGylation plays a crucial role in the application of liposomes, significantly extending their circulation half-life *in vivo*. This characteristic is attributed to the hydration layer formed by PEG molecules on the surface of the liposome, which effectively reduces the likelihood of the liposome being recognized and cleared by the immune system, thereby allowing it to maintain a stable presence in the bloodstream for a longer duration and providing a more sufficient time window for drug delivery (Tanifum et al., 2012).

In the treatment of PD, liposomes have demonstrated significant application value. Various studies have utilized liposome-based drug delivery systems for PD treatment. Stefano et al. prepared liposomes encapsulating the L-DOPA prodrug, and through *in vivo* microdialysis monitoring, it was observed that following intraperitoneal injection of these liposomes, the concentrations of L-DOPA and DA in the rat striatum significantly increased. The (+)-1b liposome formulation elevated the baseline level of DA in the rat striatal dialysate by approximately 2.5 times (Di Stefano et al., 2004). Additionally, Stefano et al. synthesized new maleic and fumaric diamide levodopa prodrugs and prepared their corresponding liposomal formulations. Studies have indicated that both (+)-4 and (+)-4 Lip can induce sustained release of DA in the striatal dialysate of rats, and compared to equimolar L-DOPA itself, they enhance the release of DA in the rat brain (Di Stefano et al., 2006). This fully demonstrates the feasibility of liposomes as drug carriers in the treatment of PD. Furthermore, liposomes have been shown to encapsulate gases, such as nitric oxide (NO) and xenon (Xe), providing a solid foundation for the development of hydrogen-loaded liposomes (Fix et al., 2015). Given the successful cases of liposome-based drug delivery for PD treatment and their capacity to encapsulate gases, it is reasonable to speculate that hydrogen-loaded liposomes could serve as an efficient method for hydrogen delivery. By leveraging the properties of liposomes, hydrogen-loaded liposomes can deliver hydrogen more effectively to the brain, enhancing both the delivery efficiency and concentration of hydrogen in this organ, thereby improving the therapeutic efficacy for PD.

## 6 Advances in hydrogen research in PD

To date, numerous experiments have been conducted on hydrogen therapy for PD. In animal studies, hydrogen has demonstrated promising therapeutic effects for PD. However, the outcomes of clinical trials on hydrogen therapy have not been as favorable as those observed in preclinical experiments.

### 6.1 Preclinical research

In preclinical studies of PD, the neuroprotective effects of hydrogen have been validated through various animal models.

In the construction of preclinical models for PD, neurotoxins are primarily utilized. Among the neurotoxin-induced models, MPTP and 6-OHDA are the most prevalent and widely applied to simulate the pathological features of acute PD.

MPTP, a neurotoxin, can freely cross the blood–brain barrier (BBB) and induce PD (PD)-like phenotypes by selectively damaging dopaminergic neurons (Schmidt and Ferger, 2001). MPTP, which selectively damages nigrostriatal dopaminergic neurons through its metabolite MPP^+^, is a commonly used model for studying the pathological mechanisms of PD. Once transported into neurons, MPP^+^ inhibits the activity of mitochondrial complex I, leading to oxidative stress that directly causes the deformation and death of DA neurons, ultimately resulting in PD (Geng et al., 2019). The MPTP model is currently the most prevalent model in PD research, effectively replicating the behavioral characteristics of PD while accurately simulating the progressive degeneration of DA neurons in the SNpc. By leveraging the inherent ability of MPTP to cross the BBB effectively, this model primarily employs various administration methods, including intraperitoneal, subcutaneous, and intramuscular injections, as well as intravenous infusion. MPTP can also be utilized to construct acute, subacute, or chronic PD models by adjusting the injection dose and frequency. However, the acute model exhibits a shorter disease course, typically characterized by rapid onset and high mortality (Xie et al., 2020). Experimental animals may develop resistance to MPTP and spontaneously return to normal within a short period, thereby failing to replicate the long-term progressive nature of human PD. In preclinical studies of hydrogen therapy for PD, all experiments utilizing MPTP modeling employed intraperitoneal injection, with only one experiment adopting the subcutaneous osmotic pump method to establish a chronic model (Fujita et al., 2009).

6-OHDA is a neurotoxin with a chemical structure similar to DA that selectively destroys DA neurons in the SNpc, ultimately leading to the death and degeneration of these neurons. However, unlike MPTP, 6-OHDA cannot cross the blood–brain barrier (BBB) (Kostrzewa and Jacobowitz, 1974). Consequently, studies utilize a brain stereotaxic instrument to stereotactically inject 6-OHDA to induce PD, with unilateral injection being the most common method for establishing the 6-OHDA model. Symptoms manifest as motor disorders on the contralateral side of the injection site, facilitating the observation of clinical intervention effects, with minimal unrelated side effects and a high survival rate (Schober, 2004). In PD animal models for hydrogen treatment, experiments using 6-OHDA have consistently employed a brain stereotaxic instrument to construct a unilateral Parkinson’s model. Notably, the formation of LB, a key pathological hallmark of PD, has not been observed in this model. Since both MPTP and 6-OHDA primarily mimic acute PD models, a lack of chronic models persists.

While these acute models can simulate the initial pathological changes of PD, they fail to comprehensively reflect the chronic progressive nature of the disease. PD is a progressive disorder characterized not only by the gradual worsening of motor symptoms but also by the emergence of non-motor symptoms. The limitations of acute models stem from their inability to mimic long-term neurodegenerative processes and adequately represent the complex pathological changes throughout the course of PD. To address these limitations, researchers have begun to utilize chronic models to more accurately simulate the natural progression of PD. Some studies have attempted to model chronic PD using subcutaneous infusion pumps. Recent research has demonstrated that AAV-α-syn or SNCA transgenic models exhibit greater clinical relevance in simulating the chronic progression of PD.

Research indicates that point mutations in the SNCA gene can induce abnormal folding and accumulation of α-syn (Abul Khair et al., 2018), thereby triggering PD. The SNCA model is currently the most widely used transgenic model for PD, characterized by its ability to exhibit α-syn aggregation. Currently, transgenic mouse models overexpressing A53T and A30P mutations are extensively utilized. The A53T transgenic mouse model effectively reflects the pathological manifestations of α-syn, demonstrating significant neurodegeneration, deterioration of motor function, and non-motor symptoms (Taguchi et al., 2020). In contrast, the A30P transgenic mouse model exhibits non-motor symptom disorders characteristic of early-stage PD in humans (Veys et al., 2021). Additionally, by overexpressing the human α-syn gene in wild-type mice, a chronic model of PD can also be established (Iba et al., 2020).

In contrast to the systemic expression of the SNCA model, AAV-α-syn is localized. AAV-α-syn refers to a construct that utilizes the Adeno-Associated Virus (AAV) as a vector to carry the α-syn gene. By injecting AAV-α-syn into the brains of animals, it induces the overexpression of α-syn (Kelly et al., 2021), thereby simulating the pathological features of human neurodegenerative diseases. Compared to neurotoxins, the AAV delivery system offers higher specificity and stability while avoiding the systemic damage caused by neurotoxins. This makes the model highly valuable for studying disease mechanisms, drug screening, and therapeutic development. However, in the AAV-CIBOP study, it was found that AAV delivery could also introduce some adverse effects, such as glial cell activation and a certain loss of specificity (Rayaprolu et al., 2022).

Current research on hydrogen therapy for PD has not utilized either AAV-α-syn or SNCA transgenic mice. Future studies should place greater emphasis on employing AAV-α-syn or SNCA transgenic models to more comprehensively simulate the chronic characteristics of PD. This approach will not only enhance our understanding of the potential role of hydrogen in the chronic progression of PD but also provide more reliable experimental evidence for clinical translational research.

The therapeutic effect of hydrogen on PD was first systematically verified in a 6-OHDA-induced rat model of PD (Fu et al., 2009). Fu et al. (2009) found that drinking hydrogen water can significantly alleviate 6-OHDA-induced nigrostriatal degeneration in rats and reduce the loss of dopaminergic cells. In this study, a PD model was constructed by stereotactically injecting 6-OHDA into the striatum of rats, and the effects of hydrogen water intervention were compared. The experiments revealed that freely drinking hydrogen water (0.4 mM) starting either 7 days before or 3 days after the model construction significantly reduced amphetamine-induced rotational behavior and protected nigral DA neurons. Although hydrogen water was ineffective against acute oxidative damage (48 h post-surgery), histological and behavioral analyses demonstrated that long-term intervention indeed delayed the progression of PD. In the same year, Fujita’s team further validated this discovery, Fujita et al. (2009) found that mice continuously consuming low-concentration hydrogen water (0.08 ppm) lost fewer nigrostriatal dopaminergic neurons compared to the control group. And the protective effect of low-concentration hydrogen water was effective, with no significantly difference compared to high-concentration hydrogen water. The study established both acute and chronic PD mouse models using MPTP, demonstrating that hydrogen water at low and high concentrations (0.08–1.5 ppm) significantly reduced dopaminergic neuron loss and decreased the accumulation of oxidative markers, such as 4-HNE and 8-oxoG. Furthermore, the research indicated that hydrogen can effectively scavenge ·OH, thereby reducing lipid peroxidation and DNA damage, although it does not have a significant effect on superoxide (O₂^−^). Both studies observed protective effects on dopaminergic neurons; despite employing different animal models, the results were consistent, thereby laying a solid foundation for the application of hydrogen in the treatment of PD. Table 5 systematically summarizes the key findings from preclinical studies on hydrogen therapy in PD.

**Table 5:** Summary of preclinical studies on hydrogen therapy for PD.

Animal/model type	Drug administration method	Concentration and predicted method of administration	Major findings	Mechanism	Ref.
6-OHDA Mouse	Oral administration of quinine	0.4 mM, ad libitum, start 7 days before modeling and continue 3 days after modeling	Reduced motor behavior, neuroprotection, and anti-inflammation	Neuroprotection, anti-inflammatory	Fu et al. (2009)
MPTP Mouse	Oral administration of quinine	0.08–1.5 ppm, ad libitum, continuous administration	Reduced DA neurodegeneration, decreased 4-HNE and 8-oxoG	Antioxidation, DNA protection	Fujita et al. (2009)
MPTP Mouse	Oral administration of quinine	0.8–1.2 ppm, ad libitum, continuous administration	Induced ghrelin secretion, neuroprotection of DA neurons	Ghrelin pathway	Matsumoto et al. (2013)
Ghrelin-KO vs. WT MPTP Mouse	Oral administration of quinine	0.8 ppm, ad libitum, continuous administration	KO mice have no ghrelin effect, mechanism by which ghrelin does not rely on	Gastrointestinal pathways, ghrelin-independent	Yoshii et al. (2017)
6-OHDA Mouse	Oral administration of quinine + inhalation of gas	1.0–1.2 ppm quinine + gas inhalation 6 h/day, continuous	Improved TH-positive cells, reduced movement disorders	Suppression of dopaminergic degeneration, alleviates motor dysfunction	Ito et al. (2012)
Rotenone-Induced Rat	Injection of quinine and salt	≥0.6 mM, intraperitoneal injection, 21 days	Improved motor function, reduced α-syn and ROS damage	PI3K/AKT/mTOR pathway activation	Zhang et al. (2021)
MPTP Mouse	Oral administration of synthetic compound	400 mL/day for 24 h, oral administration	Increased DA neurodegeneration, alleviated inflammation and motor damage	Anti-inflammatory, antioxidation	Kobayashi et al. (2020)

As research progresses, an increasing number of experiments are focusing on the role of the brain-gut axis in hydrogen therapy for PD. Matsumoto et al. (2013) constructed a PD mouse model using MPTP and found that drinking hydrogen water significantly increased ghrelin secretion. By employing a *β*₁-adrenergic receptor blocker and a ghrelin receptor antagonist, they discovered that the oral intake of hydrogen water activates the β₁-adrenergic receptor signaling pathway in the stomach, inducing ghrelin secretion, which in turn protects dopaminergic neurons in MPTP-induced PD model mice. This finding reveals a new mechanism through which hydrogen exerts neuroprotective effects indirectly via the stomach-brain axis. However, Yoshii’s study yielded different results compared to those of Matsumoto. Yoshii et al. (2017) also constructed a PD mouse model using MPTP, but utilized wild-type and Ghrelin-KO mice. By detecting TH + neurons, they found that hydrogen water still exerted a neuroprotective effect in ghrelin-deficient mice. The study also employed a ghrelin receptor antagonist, discovering that the protective effect of hydrogen disappeared in wild-type mice after blockade, but there was no significant change in KO mice. Therefore, the researchers concluded that hydrogen might exert its neuroprotective effect through a ghrelin-independent pathway, potentially involving other compensatory mechanisms. The series of studies mentioned above highlights the complexity of hydrogen’s neuroprotection and provides direction for exploring novel compensatory factors. In addition, Ito et al. (2012) further optimized the hydrogen intervention strategy by comparing the effects of hydrogen water with lactulose (a synthetic disaccharide that generates hydrogen gas through gut microbiota metabolism) in a PD model. The results revealed that the exhaled hydrogen concentration was significantly higher in the hydrogen water group the induction rotation test and the count of tyrosine hydroxylase (TH) positive cells revealed that both the hydrogen water group and the intermittent hydrogen inhalation group exhibited significant therapeutic effects. In contrast, the continuous hydrogen group and the lactulose group did not demonstrate any therapeutic efficacy. Consequently, the researchers speculate that the protective effect of hydrogen may be contingent upon the pulsed exposure mode and the regulation of signaling pathways, rather than solely dependent on the quantity of hydrogen produced or the duration of continuous exposure. The study also indicated that the signal-regulating activity of hydrogen may be a key mechanism underlying its protective effect against PD. In their 2021 study, Zhang et al. (2021) clearly indicated that hydrogen-saturated saline mediates neuroprotection through autophagy via the PI3K/AKT/mTOR pathway during the early and medium stages of rotenone-induced PD in rats. The study employed rotenone (3 mg/kg/day for 21 days) administered via intraperitoneal injection to establish a rat model of PD, with hydrogen-saturated saline (≥0.6 mM) applied at different stages (early, middle, late) for treatment. The results demonstrated that early and middle-stage treatment with hydrogen-saturated saline significantly improved cardiovascular and motor dysfunction, reduced neuronal loss, ROS, and α-syn accumulation, and activated autophagy by inhibiting the PI3K/AKT/mTOR pathway. Late-stage intervention showed no significant effects, suggesting that hydrogen therapy should be initiated in the early pathological stages of PD.

With the advancement of technology, researchers have begun to prepare hydrogen-producing nanomaterials for the treatment of PD. Kobayashi et al. (2020) developed a silicon-based agent that, upon oral intake as part of a diet, continuously generates hydrogen in the intestines at a rate of 400 mL/g·24 h. Experiments have shown that this silicon-based agent significantly reduces the expression of oxidative stress markers (8-OHdG), inflammatory factors (IL-6, CCL2), and apoptosis-related genes (caspase-3), thereby alleviating damage to dopamine neurons.

Although multiple mechanisms have been identified, the interplay and collaboration among these pathways remain unclear. For instance, hydrogen remains effective in ghrelin knockout mice, suggesting the existence of unknown compensatory pathways; however, the pathological differences between animal models and human PD may limit clinical translation.

Moreover, it is widely recognized that patients with PD often experience significant non-motor symptoms, including gastrointestinal dysfunction and sleep disorders. Current research on the therapeutic effects of hydrogen indicates that molecular hydrogen has made notable progress in alleviating constipation and sleep disturbances.

Preclinical studies have demonstrated that molecular hydrogen alleviates constipation by modulating the gut microbiota and reducing oxidative stress. Chen et al. (2024) investigated the effects of hydrogen-rich water (HRW) using a loperamide-induced constipation rat model. The study found that hydrogen-rich water (>3.0 ppm) significantly improved bowel movements in constipated rats. Analysis of 16S rDNA gene sequencing revealed that hydrogen-rich water modulated the gut microbiota, increasing the abundance of beneficial bacteria and decreasing the levels of harmful bacteria associated with inflammation. Further analysis indicated that hydrogen-rich water significantly decreased the levels of ROS in the colon tissues of constipated rats, reduced malondialdehyde (MDA) content, and enhanced superoxide dismutase (SOD) activity, thereby alleviating intestinal oxidative stress. The study also found that hydrogen-rich water alleviates intestinal oxidative stress by upregulating the expression of SIRT1, Nrf2, and HO-1, thus activating the SIRT1/Nrf2/HO-1 signaling pathway, which further improves constipation. In summary, hydrogen effectively alleviates constipation symptoms through intestinal microbiota regulation and antioxidant effects, providing strong preclinical evidence for its use as a novel strategy in constipation treatment.

Mizuno et al. (2017) investigated the effects of hydrogen-rich water (HRW) on sleep quality in adults through a double-blind, placebo-controlled study design. The study found that the Pittsburgh Sleep Quality Index (PSQI) scores in the HRW group significantly decreased after the intervention compared to before the intervention, indicating a positive impact on sleep quality improvement. Additionally, the power of the low-frequency component (LF) in the resting state significantly decreased in the HRW group, suggesting that HRW may enhance autonomic nervous function by reducing sympathetic nervous activity, which is closely related to improved sleep quality. Although the PSQI score of the HRW group significantly decreased post-intervention, the comparison with the placebo group did not reach statistical significance, potentially due to the placebo effect and the small sample size affecting statistical power. Nonetheless, the improvement in sleep quality attributed to HRW is noteworthy. This result provides preliminary clinical evidence for the potential role of hydrogen in alleviating sleep disorders, although further research is necessary to verify its specific effects and mechanisms. Additionally, in a follow-up study on hydrogen therapy in advanced cancer patients, it was found that after 2 weeks of hydrogen inhalation therapy, the insomnia symptoms of advanced cancer patients significantly improved, indicating that hydrogen therapy has a beneficial effect on sleep disorders. This finding supports the potential of hydrogen in addressing sleep issues and other related aspects (Chen et al., 2019).

### 6.2 Clinical research

H_2_ has demonstrated antioxidant potential in preclinical studies, and several clinical investigations have explored its application in PD. Nonetheless, the clinical evidence remains inconsistent, as some studies report no significant improvement in symptoms. This variability in outcomes highlights the necessity for more rigorous clinical trials to establish its efficacy. Current clinical evidence indicates that hydrogen therapy is notable for its safety; nonetheless, there remains a lack of consensus regarding its effectiveness in achieving symptomatic improvement in PD.

Early small-scale trials suggested potential benefits of hydrogen therapy. A randomized, double-blind trial conducted by Yoritaka et al. (2013) found that after 48 weeks of daily consumption of 1.5 ppm hydrogen water by patients with PD, the total score on the Unified PD Rating Scale (UPDRS) significantly improved in the hydrogen group. Notably, no serious adverse effects were reported during the trial. This indicates that hydrogen water may provide symptomatic relief for early-stage PD patients, suggesting its potential as a therapeutic adjunct. However, subsequent larger studies failed to replicate these results (Yoritaka et al., 2016). A multicenter, randomized, double-blind trial further validated the safety of long-term hydrogen water consumption. However, it revealed no statistically significant difference in the change in UPDRS total score between the hydrogen water group and the placebo group at 72 weeks (Yoritaka et al., 2016). Notably, patients in this trial experienced significantly less worsening of UPDRS scores compared to historical controlled studies. This suggests a potential weak delaying effect of hydrogen water on disease progression, however, further validation is required alongside more sensitive biomarkers.

A randomized controlled trial investigating the combination of hydrogen water and dopas hydrazine demonstrated a significant reduction in UPDRS scores and an improvement in activities of daily living (ADL) scores after 3 months of treatment. This supports the potential of hydrogen as an effective adjunct to dopaminergic medications. These findings suggest that hydrogen water may enhance the symptomatic effects of conventional dopaminergic therapy. However, the study did not evaluate long-term effects, and further research is necessary to establish its clinical relevance (Wang and Xu, 2021).

Furthermore, an open-label, phase I/IIa study investigated the combination of hydrogen water and photobiomodulation (PBM) therapy, demonstrating a significant reduction in Unified PD Rating Scale (UPDRS) scores after 1e week of treatment. This further highlights the potential of hydrogen to enhance the effects of other therapeutic interventions. The results suggest that hydrogen may exert neuroprotective effects not only as a standalone therapy but also as an adjunctive treatment. This underscores the necessity for further research into combination therapies that incorporate hydrogen as a potential enhancer of other neuroprotective interventions (Hong et al., 2021). Additionally, exploration of administration methods other than hydrogen water consumption has produced mixed results.

A randomized, double-blind, placebo-controlled trial evaluated the long-term inhalation of hydrogen (6.5% H₂, twice daily for 1 h each session, over 16 weeks). No significant difference in UPDRS scores was observed between the hydrogen group and the placebo group. This finding further supports the conclusion that, under the current study conditions, hydrogen inhalation has not yet demonstrated a clear effect on symptom improvement (Yoritaka et al., 2021). However, in another study, a randomized crossover trial conducted by Hirayama et al. involving 20 patients with PD found that twice-daily inhalation of 1.2–1.4% hydrogen over 4 weeks did not lead to improvements in UPDRS scores, olfactory function, or the ability to perform activities of daily living. However, the study did reveal a significant increase urinary levels of the oxidative stress marker 8-OHdG (Yoritaka et al., 2018). The investigators hypothesized that hydrogen might indirectly exert neuroprotective effects by inducing hormonal responses associated with mild oxidative stress, potentially mediated through the activation of the Nrf2 pathway and the pro-inflammatory factor NF-κB. Nonetheless, the short-term intervention did not translate into clinical symptom improvement. Table 6 summarizes the key clinical trials investigating hydrogen therapy in PD. It provides a comparative analysis of each study’s research design, experimental procedures, experimental groups, sample sizes, endpoints, limitations, and results.

**Table 6:** Summary of clinical trials investigating hydrogen therapy in PD.

Study design	Intervention	Groups	Primary endpoint	Limitations	Results	Ref.
Randomized, double-blind, placebo-controlled trial	Hydrogen-rich water (1,000 mL/day, 0.8 mM) vs. placebo (plain water) for 48 weeks	Hydrogen group (*n* = 9); placebo group (*n* = 9)	Change in UPDRS total score	Small sample size; short duration; limited to levodopa-treated patients	Significant UPDRS improvement in hydrogen group; placebo group deteriorated. Safe with no adverse events.	Yoritaka et al. (2013)
Randomized, double-blind, placebo-controlled trial	Hydrogen inhalation (6.5% in air, 1 h twice daily) vs. placebo for 16 weeks	Hydrogen group (*n* = 7); placebo group (*n* = 8)	Change in MDS-UPDRS total score	Small sample size; excluded advanced PD patients; low protocol adherence	No significant differences in MDS-UPDRS or biomarkers. Safe with no adverse events.	Yoritaka et al. (2021)
Randomized, double-blind, placebo-controlled trial	Hydrogen-rich water (1,000 mL/day) vs. placebo for 72 weeks	Hydrogen group; placebo group (93 female, 85 male)	Change in UPDRS total score	Excluded prodromal and advanced PD patients; placebo water had low hydrogen concentration	No significant differences in UPDRS or PDQ-39. Safe with no serious adverse events.	Yoritaka et al. (2016)
Open-label, single-arm, phase I/IIa study	Combined PBM (near-infrared light, 30 min daily) and hydrogen-rich water (200 mL/day, 2.5 ppm) for 2 weeks	Single-arm (*n* = 18; 17 completed)	Change in UPDRS total score	Small sample size; no control group; short duration; open-label design	Significant UPDRS improvement sustained post-treatment. Safe with no adverse events.	Hong et al. (2021)
Randomized clinical study	Hydrogen-rich water (800–1,000 mL/day) vs. purified water, both with dopas hydrazine for 3 months	Hydrogen group (*n* = 32); control group (*n* = 32)	Change in UPDRS and ADL scores	Small sample size; unknown long-term effects	Significant UPDRS improvement in hydrogen group (*p* < 0.05).	Wang and Xu, (2021)
Randomized, double-blind, placebo-controlled crossover trial	Hydrogen inhalation (1.2–1.4% in air, 10 min twice daily) vs. placebo for 4 weeks	Hydrogen group; control group	Olfactory function (OSIT-J)	Small sample size; short duration; low hydrogen concentration	No significant changes in olfactory function or biomarkers.	Yoritaka et al. (2018)

The ambivalence of clinical results may be closely related to several factors: (1) differences in patient staging, as positive trials predominantly included patients with early-stage PD, while negative trials encompassed a broader range of disease stages, where neuronal damage may have become irreversible in late-stage patients (Yoritaka et al., 2018); (2) variations in hydrogen concentration and the duration of the intervention, as indicated by Yoritaka et al. (2017) study protocol, which demonstrated that the concentration and duration of hydrogen water consumption significantly influenced efficacy assessment. Conversely, the inhalation trial’s exposure of only 20 min per day may not be sufficient to achieve an effective central concentration (Yoritaka et al., 2016; Yoritaka et al., 2018); and (3) limitations of evaluation metrics, as the total score of the UPDRS may lack sensitivity to subtle functional changes. Future studies should incorporate cerebrospinal fluid α-syn, DA transporter PET imaging, and other multidimensional indicators to enable a more comprehensive and sensitive evaluation (Yoritaka et al., 2018).

## 7 Discussion

The therapeutic potential of molecular hydrogen in PD and other neurodegenerative disorders has been progressively elucidated through multidimensional studies. At the mechanistic level, H₂ exerts neuroprotective effects through three core mechanisms: (1) selective scavenging of toxic ROS, such as -OH and ONOO-, along with the activation of the Nrf2/ARE pathway to enhance endogenous antioxidant defenses; (2) inhibition of microglial M1 polarization and NLRP3 inflammasome activation, leading to a reduction in pro-inflammatory factors such as IL-1β and TNF-α; (3) modulation of the Bax/Bcl-2 balance and the caspase cascade, as well as the blockade of mitochondria-dependent apoptotic pathways. Animal studies have confirmed that H₂ enhances the survival of dopaminergic neurons in PD models, and significantly improves rotational behavior and motor coordination. In terms of clinical translation, although current results indicate variability in the improvement of UPDRS scores with hydrogen interventions, such as hydrogen-rich water or inhalation, its safety profile underscores its unique potential as an adjunctive therapy. It is important to note that the variability in clinical outcomes may be attributed to several factors, including study design, sample size, and differences in the disease stages of patient populations. Current clinical evidence remains relatively weak, with only one study demonstrating that hydrogen therapy alone can improve UPDRS scores in PD (Yoritaka et al., 2013), while two additional studies suggest that hydrogen combined with other treatments may provide benefits for PD patients. However, several other studies have failed to show significant improvements. Despite these inconsistencies, the safety profile of hydrogen therapy appears robust, with no major adverse effects reported in clinical trials. This indicates that hydrogen therapy has potential for use in early-stage PD patients, particularly regarding neuroprotection and the slowing of disease progression. For future clinical applications, large-scale and long-term studies are necessary to establish more consistent clinical outcomes and validate the actual efficacy of hydrogen therapy. Future research should also investigate the impact of hydrogen therapy on non-motor symptoms of PD, particularly cognitive decline and sleep disturbances, which are often inadequately addressed by traditional treatments. Furthermore, given the antioxidant and anti-inflammatory properties of hydrogen, it may not only be beneficial for motor symptoms but also play a positive role in slowing the chronic progression of PD and enhancing patients’ quality of life.

In conclusion, while the current clinical evidence is not yet sufficient, the safety and preliminary efficacy of hydrogen suggest that it may serve as a potential adjunct therapy that warrants further validation in future clinical studies. To enhance the clinical application of hydrogen therapy, it is essential to conduct more multicenter, large-sample, long-term follow-up studies, particularly to investigate its efficacy in patient populations at various stages of disease progression.

Furthermore, considering the limitations of current monotherapies, molecular hydrogen may serve as a promising adjuvant therapy in PD. Long-term levodopa treatment, while effective in alleviating motor symptoms, has been associated with increased oxidative stress and the generation of toxic metabolites, which can accelerate the loss of dopaminergic neurons. The selective antioxidant effect of H₂, particularly its ability to neutralize ·OH without disrupting physiological ROS, may mitigate levodopa-induced neurotoxicity and thus prolong therapeutic efficacy. In addition, deep brain stimulation (DBS), though effective in controlling motor symptoms, may cause local inflammation and gliosis due to electrode implantation. Preclinical evidence suggests that H₂‘s anti-inflammatory and neuroprotective properties may help attenuate such adverse responses, although this requires further clinical validation. These potential synergies indicate that H₂ could be strategically integrated as an adjunct to existing treatment regimens, enhancing neuroprotection and functional outcomes.

Despite the preliminary validation of molecular hydrogen’s neuroprotective effects, several key issues require further investigation. The standardization of dosing regimens is essential; current methods of hydrogen delivery, including inhalation and hydrogen-enriched water, exhibit significant variability in hydrogen concentration within brain tissues. Pharmacokinetic studies are necessary to elucidate the effects of different administration modalities on hydrogen accumulation in nigral regions (Liu et al., 2021). Furthermore, the multi-target mechanisms of H_2_ need to be more thoroughly explored. While hydrogen may exert its effects through hormonal pathways such as free radical scavenging, inflammation inhibition, and regulating ghrelin, its regulatory impact on α-syn nucleoprotein aggregation warrants in-depth investigation in conjunction with proteomic analyses (Matsumoto et al., 2013). Notably, the potential of hydrogen interventions to alleviate non-motor symptoms, such as constipation and sleep disorders, may address the limitations of existing therapies (Pluta et al., 2022).

## References

[ref1] Abul Khair S. B. Dhanushkodi N. R. Ardah M. T. Chen W. Yang Y. Haque M. E. (2018). Silencing of Glucocerebrosidase gene in Drosophila enhances the aggregation of Parkinson's disease associated α-Synuclein mutant A53T and affects locomotor activity. Front. Neurosci. 12:81. doi: 10.3389/fnins.2018.00081, PMID: 29503608 PMC5820349

[ref2] Arena G. Gelmetti V. Torosantucci L. Vignone D. Lamorte G. De Rosa P.. (2013). PINK1 protects against cell death induced by mitochondrial depolarization, by phosphorylating Bcl-xL and impairing its pro-apoptotic cleavage. Cell Death Differ. 20, 920–930. doi: 10.1038/cdd.2013.19, PMID: 23519076 PMC3679455

[ref3] Badanjak K. Fixemer S. Smajić S. Skupin A. Grünewald A. (2021). The contribution of microglia to Neuroinflammation in Parkinson's disease. Int. J. Mol. Sci. 22:676. doi: 10.3390/ijms22094676, PMID: 33925154 PMC8125756

[ref4] Bedard K. Krause K. H. (2007). The NOX family of ROS-generating NADPH oxidases: physiology and pathophysiology. Physiol. Rev. 87, 245–313. doi: 10.1152/physrev.00044.2005, PMID: 17237347

[ref5] Bernal-Conde L. D. Ramos-Acevedo R. Reyes-Hernández M. A. Balbuena-Olvera A. J. Morales-Moreno I. D. Argüero-Sánchez R.. (2019). Alpha-Synuclein physiology and pathology: a perspective on cellular structures and organelles. Front. Neurosci. 13:1399. doi: 10.3389/fnins.2019.01399, PMID: 32038126 PMC6989544

[ref6] Betarbet R. Sherer T. B. MacKenzie G. Garcia-Osuna M. Panov A. V. Greenamyre J. T. (2000). Chronic systemic pesticide exposure reproduces features of Parkinson's disease. Nat. Neurosci. 3, 1301–1306. doi: 10.1038/81834, PMID: 11100151

[ref7] Bond A. E. Shah B. B. Huss D. S. Dallapiazza R. F. Warren A. Harrison M. B.. (2017). Safety and efficacy of focused ultrasound Thalamotomy for patients with medication-refractory, tremor-dominant Parkinson disease: a randomized clinical trial. JAMA Neurol. 74, 1412–1418. doi: 10.1001/jamaneurol.2017.3098, PMID: 29084313 PMC5822192

[ref8] Bové J. Martínez-Vicente M. Dehay B. Perier C. Recasens A. Bombrun A.. (2014). BAX channel activity mediates lysosomal disruption linked to Parkinson disease. Autophagy 10, 889–900. doi: 10.4161/auto.28286, PMID: 24686337 PMC5119069

[ref9] Buchholz B. M. Kaczorowski D. J. Sugimoto R. Yang R. Wang Y. Billiar T. R.. (2008). Hydrogen inhalation ameliorates oxidative stress in transplantation induced intestinal graft injury. Am. J. Transplant. Off. J. Am. Soc. Transplant. Am. Soc. Transplant Surg. 8, 2015–2024. doi: 10.1111/j.1600-6143.2008.02359.x, PMID: 18727697

[ref10] Bugarski-Kirola D. Nunez R. Odetalla R. Liu I. Y. Turner M. E. (2022). Effects of adjunctive pimavanserin and current antipsychotic treatment on QT interval prolongation in patients with schizophrenia. Front. Psych. 13:892199. doi: 10.3389/fpsyt.2022.892199, PMID: 36147980 PMC9486460

[ref11] Cadenas B. Fita-Torró J. Bermúdez-Cortés M. Hernandez-Rodriguez I. Fuster J. L. Llinares M. E.. (2019). L-ferritin: one gene, five diseases; from hereditary Hyperferritinemia to Hypoferritinemia-report of new cases. Pharmaceuticals 12:17. doi: 10.3390/ph12010017 30678075 PMC6469184

[ref12] Calabresi P. Standaert D. G. (2019). Dystonia and levodopa-induced dyskinesias in Parkinson's disease: is there a connection? Neurobiol. Dis. 132:104579. doi: 10.1016/j.nbd.2019.104579, PMID: 31445160 PMC6834901

[ref13] Calvello R. Cianciulli A. Nicolardi G. De Nuccio F. Giannotti L. Salvatore R.. (2017). Vitamin D treatment attenuates Neuroinflammation and dopaminergic neurodegeneration in an animal model of Parkinson's disease, shifting M1 to M2 microglia responses. J. Neuroimmune Pharmacol. 12, 327–339. doi: 10.1007/s11481-016-9720-7, PMID: 27987058

[ref14] Chang M. C. Kwak S. G. Kwak S. (2021). Effect of dietary vitamins C and E on the risk of Parkinson's disease: a meta-analysis. Clini. Nutri. 40, 3922–3930. doi: 10.1016/j.clnu.2021.05.011, PMID: 34139465

[ref15] Chen W. Jiang S. Li S. Li C. Xu R. (2024). OSMR is a potential driver of inflammation in amyotrophic lateral sclerosis. Neural Regen. Res. 19, 2513–2521. doi: 10.4103/1673-5374.391309, PMID: 38526287 PMC11090450

[ref16] Chen J. B. Kong X. F. Lv Y. Y. Qin S. C. Sun X. J. Mu F.. (2019). "real world survey" of hydrogen-controlled cancer: a follow-up report of 82 advanced cancer patients. Med. Gas Res. 9, 115–121. doi: 10.4103/2045-9912.266985, PMID: 31552873 PMC6779007

[ref17] Chen K. D. Wang K. L. Chen C. Zhu Y. J. Tang W. W. Wang Y. J.. (2024). Hydrogen-rich water alleviates constipation by attenuating oxidative stress through the sirtuin1/nuclear factor-erythroid-2-related factor 2/heme oxygenase-1 signaling pathway. World J. Gastroenterol. 30, 2709–2725. doi: 10.3748/wjg.v30.i20.2709, PMID: 38855154 PMC11154682

[ref18] Chen K. Wang H. Ilyas I. Mahmood A. Hou L. (2023). Microglia and astrocytes dysfunction and key Neuroinflammation-based biomarkers in Parkinson's disease. Brain Sci. 13:634. doi: 10.3390/brainsci13040634, PMID: 37190599 PMC10136556

[ref19] Chittasupho C. Tadtong S. Vorarat S. Imaram W. Athikomkulchai S. Samee W.. (2022). Development of jelly loaded with Nanogel containing natural L-Dopa from *Mucuna pruriens* seed extract for neuroprotection in Parkinson's disease. Pharmaceutics 14:79. doi: 10.3390/pharmaceutics14051079, PMID: 35631666 PMC9147856

[ref20] Cordeiro D. Stern T. Stern S. (2024). Focusing on the tetra-partite synapse in Parkinson's disease research using human patient-derived neurons. Neural Regen. Res. 19, 979–981. doi: 10.4103/1673-5374.382235, PMID: 37862197 PMC10749603

[ref21] Di Rita A. D'Acunzo P. Simula L. Campello S. Strappazzon F. Cecconi F. (2018). AMBRA1-mediated Mitophagy counteracts oxidative stress and apoptosis induced by neurotoxicity in human neuroblastoma SH-SY5Y cells. Front. Cell. Neurosci. 12:92. doi: 10.3389/fncel.2018.00092, PMID: 29755319 PMC5932353

[ref22] Di Stefano A. Carafa M. Sozio P. Pinnen F. Braghiroli D. Orlando G.. (2004). Evaluation of rat striatal L-dopa and DA concentration after intraperitoneal administration of L-dopa prodrugs in liposomal formulations. J. Control. 99, 293–300. doi: 10.1016/j.jconrel.2004.07.010, PMID: 15380638

[ref23] Di Stefano A. Sozio P. Iannitelli A. Marianecci C. Santucci E. Carafa M. (2006). Maleic- and fumaric-diamides of (O,O-diacetyl)-L-Dopa-methylester as anti-Parkinson prodrugs in liposomal formulation. J. Drug Target. 14, 652–661. doi: 10.1080/10611860600916636, PMID: 17090401

[ref24] Dobkin R. D. Menza M. Allen L. A. Gara M. A. Mark M. H. Tiu J.. (2011). Cognitive-behavioral therapy for depression in Parkinson's disease: a randomized, controlled trial. Am. J. Psychiatry 168, 1066–1074. doi: 10.1176/appi.ajp.2011.10111669, PMID: 21676990 PMC3186855

[ref25] Dole M. Wilson F. R. Fife W. P. (1975). Hyperbaric hydrogen therapy: a possible treatment for cancer. Science 190, 152–154. doi: 10.1126/science.1166304, PMID: 1166304

[ref26] Elkouzi A. Vedam-Mai V. Eisinger R. S. Okun M. S. (2019). Emerging therapies in Parkinson disease - repurposed drugs and new approaches. Nat. Rev. Neurol. 15, 204–223. doi: 10.1038/s41582-019-0155-7, PMID: 30867588 PMC7758837

[ref27] Erlich J. R. To EE Luong R. Liong F. Liong S. Oseghale O.. (2022). Glycolysis and the pentose phosphate pathway promote LPS-induced NOX2 oxidase- and IFN-β-dependent inflammation in macrophages. Antioxidants 11:1488. doi: 10.3390/antiox11081488 36009206 PMC9405479

[ref28] Fenoy A. J. Simpson R. K. Jr. (2014). Risks of common complications in deep brain stimulation surgery: management and avoidance. J. Neurosurg. 120, 132–139. doi: 10.3171/2013.10.JNS131225, PMID: 24236657

[ref29] Fix S. M. Borden M. A. Dayton P. A. (2015). Therapeutic gas delivery via microbubbles and liposomes. Journal Control. Releas. 209, 139–149. doi: 10.1016/j.jconrel.2015.04.027, PMID: 25913365

[ref30] Fouke K. E. Wegman M. E. Weber S. A. Brady E. B. Román-Vendrell C. Morgan J. R. (2021). Synuclein regulates synaptic vesicle clustering and docking at a vertebrate synapse. Front. Cell Dev. Biol. 9:774650. doi: 10.3389/fcell.2021.774650, PMID: 34901020 PMC8660973

[ref31] Fox S. H. Katzenschlager R. Lim S. Y. Barton B. de Bie R. M. A. Seppi K.. (2018). International Parkinson and movement disorder society evidence-based medicine review: update on treatments for the motor symptoms of Parkinson's disease. Mov. Disord. 33, 1248–1266. doi: 10.1002/mds.27372, PMID: 29570866

[ref32] Fu Y. Ito M. Fujita Y. Ito M. Ichihara M. Masuda A.. (2009). Molecular hydrogen is protective against 6-hydroxydopamine-induced nigrostriatal degeneration in a rat model of Parkinson's disease. Neurosci. Lett. 453, 81–85. doi: 10.1016/j.neulet.2009.02.016, PMID: 19356598

[ref33] Fujita K. Seike T. Yutsudo N. Ohno M. Yamada H. Yamaguchi H.. (2009). Hydrogen in drinking water reduces dopaminergic neuronal loss in the 1-methyl-4-phenyl-1,2,3,6-tetrahydropyridine mouse model of Parkinson's disease. PLoS One 4:e7247. doi: 10.1371/journal.pone.0007247, PMID: 19789628 PMC2747267

[ref34] Fukuda K. Asoh S. Ishikawa M. Yamamoto Y. Ohsawa I. Ohta S. (2007). Inhalation of hydrogen gas suppresses hepatic injury caused by ischemia/reperfusion through reducing oxidative stress. Biochem. Biophys. Res. Commun. 361, 670–674. doi: 10.1016/j.bbrc.2007.07.088, PMID: 17673169

[ref35] Geng J. Liu W. Gao J. Jiang C. Fan T. Sun Y.. (2019). Andrographolide alleviates parkinsonism in MPTP-PD mice via targeting mitochondrial fission mediated by dynamin-related protein 1. Br. J. Pharmacol. 176, 4574–4591. doi: 10.1111/bph.14823, PMID: 31389613 PMC6932945

[ref36] Gremke N. Griewing S. Printz M. Kostev K. Wagner U. Kalder M. (2022). Association between Parkinson's disease medication and the risk of lower urinary tract infection (LUTI): a retrospective cohort study. J. Clin. Med. 11:77. doi: 10.3390/jcm11237077, PMID: 36498652 PMC9737110

[ref37] Gu Y. Huang C. S. Inoue T. Yamashita T. Ishida T. Kang K. M.. (2010). Drinking hydrogen water ameliorated cognitive impairment in senescence-accelerated mice. J. Clin. Biochem. Nutr. 46, 269–276. doi: 10.3164/jcbn.10-19, PMID: 20490324 PMC2872234

[ref38] Gu Y. Ma J. Fu Z. Xu Y. Gao B. Yao J.. (2019). Development of novel liposome-encapsulated Combretastatin A4 Acylated derivatives: prodrug approach for improving antitumor efficacy. Int. J. Nanomedicine 14, 8805–8818. doi: 10.2147/IJN.S210938, PMID: 31806973 PMC6844228

[ref39] Guo C. Chen X. Xiong P. (2014). Baicalin suppresses iron accumulation after substantia nigra injury: relationship between iron concentration and transferrin expression. Neural Regen. Res. 9, 630–636. doi: 10.4103/1673-5374.130108, PMID: 25206866 PMC4146239

[ref40] Guo X. Han C. Ma K. Xia Y. Wan F. Yin S.. (2019). Hydralazine protects nigrostriatal dopaminergic neurons from MPP(+) and MPTP induced neurotoxicity: roles of Nrf2-ARE signaling pathway. Front. Neurol. 10:271. doi: 10.3389/fneur.2019.00271 30949126 PMC6435581

[ref41] Hacker M. L. DeLong M. R. Turchan M. Heusinkveld L. E. Ostrem J. L. Molinari A. L.. (2018). Effects of deep brain stimulation on rest tremor progression in early stage Parkinson disease. Neurology 91, e463–e471. doi: 10.1212/WNL.0000000000005903, PMID: 29959266 PMC6093763

[ref42] Haque M. E. Akther M. Azam S. Kim I. S. Lin Y. Lee Y. H.. (2022). Targeting α-synuclein aggregation and its role in mitochondrial dysfunction in Parkinson's disease. Br. J. Pharmacol. 179, 23–45. doi: 10.1111/bph.15684, PMID: 34528272

[ref43] He D. Huang B. Fu S. Li Y. Ran X. Liu Y.. (2018). Tubeimoside I protects dopaminergic neurons against inflammation-mediated damage in lipopolysaccharide (LPS)-evoked model of Parkinson's disease in rats. Int. J. Mol. Sci. 19:242. doi: 10.3390/ijms19082242, PMID: 30065205 PMC6121380

[ref44] He J. Liu F. Xu T. Ma J. Yu H. Zhao J.. (2023). The role of hydrogen therapy in Alzheimer's disease management: insights into mechanisms, administration routes, and future challenges. Biomed. Pharmacother. Pharmac. 168:115807. doi: 10.1016/j.biopha.2023.115807, PMID: 37913734

[ref45] He J. Xu P. Xu T. Yu H. Wang L. Chen R.. (2024). Therapeutic potential of hydrogen-rich water in zebrafish model of Alzheimer's disease: targeting oxidative stress, inflammation, and the gut-brain axis. Front. Aging Neurosci. 16:1515092. doi: 10.3389/fnagi.2024.1515092, PMID: 39839307 PMC11746902

[ref46] Heikkila R. E. Sieber B. A. Manzino L. Sonsalla P. K. (1989). Some features of the nigrostriatal dopaminergic neurotoxin 1-methyl-4-phenyl-1,2,3,6-tetrahydropyridine (MPTP) in the mouse. Mol. Chem. Neuropathol. 10, 171–183. doi: 10.1007/BF03159727 2669769

[ref47] Herz D. M. Haagensen B. N. Christensen M. S. Madsen K. H. Rowe J. B. Løkkegaard A.. (2015). Abnormal dopaminergic modulation of striato-cortical networks underlies levodopa-induced dyskinesias in humans. Brain 138, 1658–1666. doi: 10.1093/brain/awv096, PMID: 25882651 PMC4614130

[ref48] Hong C. T. Hu C. J. Lin H. Y. Wu D. (2021). Effects of concomitant use of hydrogen water and photobiomodulation on Parkinson disease: a pilot study. Medicine 100:e24191. doi: 10.1097/MD.0000000000024191, PMID: 33530211 PMC7850666

[ref49] Hong Y. Shao A. Wang J. Chen S. Wu H. McBride D. W.. (2014). Neuroprotective effect of hydrogen-rich saline against neurologic damage and apoptosis in early brain injury following subarachnoid hemorrhage: possible role of the Akt/GSK3β signaling pathway. PLoS One 9:e96212. doi: 10.1371/journal.pone.0096212, PMID: 24763696 PMC3999200

[ref50] Hu D. Kabayama S. Watanabe Y. Cui Y. (2024). Health benefits of electrolyzed hydrogen water: antioxidant and anti-inflammatory effects in living organisms. Antioxidants 13:313. doi: 10.3390/antiox13030313 38539846 PMC10967432

[ref51] Hu Y. Wang P. Han K. (2022). Hydrogen attenuated inflammation response and oxidative in hypoxic ischemic encephalopathy via Nrf2 mediated the inhibition of NLRP3 and NF-κB. Neuroscience 485, 23–36. doi: 10.1016/j.neuroscience.2021.12.024, PMID: 34953939

[ref52] Iba M. Kim C. Florio J. Mante M. Adame A. Rockenstein E.. (2020). Role of alterations in protein kinase p38γ in the pathogenesis of the synaptic pathology in dementia with Lewy bodies and α-Synuclein transgenic models. Front. Neurosci. 14:286. doi: 10.3389/fnins.2020.00286, PMID: 32296304 PMC7138105

[ref53] Indrieri A. Pizzarelli R. Franco B. De Leonibus E. (2020). Dopamine, alpha-Synuclein, and mitochondrial dysfunctions in parkinsonian eyes. Front. Neurosci. 14:567129. doi: 10.3389/fnins.2020.567129, PMID: 33192254 PMC7604532

[ref54] Innamorato N. G. Jazwa A. Rojo A. I. García C. Fernández-Ruiz J. Grochot-Przeczek A.. (2010). Different susceptibility to the Parkinson's toxin MPTP in mice lacking the redox master regulator Nrf2 or its target gene heme oxygenase-1. PLoS One 5:e11838. doi: 10.1371/journal.pone.0011838, PMID: 20676377 PMC2911386

[ref55] Isik S. Yeman Kiyak B. Akbayir R. Seyhali R. Arpaci T. (2023). Microglia mediated Neuroinflammation in Parkinson's disease. Cells 12:12. doi: 10.3390/cells12071012, PMID: 37048085 PMC10093562

[ref56] Ito M. Hirayama M. Yamai K. Goto S. Ito M. Ichihara M.. (2012). Drinking hydrogen water and intermittent hydrogen gas exposure, but not lactulose or continuous hydrogen gas exposure, prevent 6-hydorxydopamine-induced Parkinson’s disease in rats. Med. Gas Res. 2:15. doi: 10.1186/2045-9912-2-15, PMID: 22608009 PMC3407490

[ref57] Itoh T. Fujita Y. Ito M. Masuda A. Ohno K. Ichihara M.. (2009). Molecular hydrogen suppresses FcepsilonRI-mediated signal transduction and prevents degranulation of mast cells. Biochem. Biophys. Res. Commun. 389, 651–656. doi: 10.1016/j.bbrc.2009.09.047 19766097

[ref58] Jakel R. J. Townsend J. A. Kraft A. D. Johnson J. A. (2007). Nrf2-mediated protection against 6-hydroxydopamine. Brain Res. 1144, 192–201. doi: 10.1016/j.brainres.2007.01.131, PMID: 17336276 PMC2062573

[ref59] Jansen R. L. Brogan B. Whitworth A. J. Okello E. J. (2014). Effects of five Ayurvedic herbs on locomotor behaviour in a *Drosophila melanogaster* Parkinson's disease model. Phytotherapy Res. 28, 1789–1795. doi: 10.1002/ptr.5199, PMID: 25091506 PMC4310928

[ref60] Jiang X. Niu X. Guo Q. Dong Y. Xu J. Yin N.. (2019). FoxO1-mediated autophagy plays an important role in the neuroprotective effects of hydrogen in a rat model of vascular dementia. Behav. Brain Res. 356, 98–106. doi: 10.1016/j.bbr.2018.05.023, PMID: 29885845

[ref61] Jiang H. Qian Z. M. Xie J. X. (2003). Increased DMT1 expression and iron content in MPTP-treated C57BL/6 mice. Acta Physiol. Sinica 55, 571–576, PMID: 14566406

[ref62] Katoh Y. Itoh K. Yoshida E. Miyagishi M. Fukamizu A. Yamamoto M. (2001). Two domains of Nrf2 cooperatively bind CBP, a CREB binding protein, and synergistically activate transcription. Genes Cells 6, 857–868. doi: 10.1046/j.1365-2443.2001.00469.x, PMID: 11683914

[ref63] Kelly R. Cairns A. G. Ådén J. Almqvist F. Bemelmans A. P. Brouillet E.. (2021). The small molecule alpha-Synuclein aggregator, FN075, enhances alpha-Synuclein pathology in subclinical AAV rat models. Biomol. Ther. 11:685. doi: 10.3390/biom11111685, PMID: 34827685 PMC8615715

[ref64] Kirkley K. S. Popichak K. A. Hammond S. L. Davies C. Hunt L. Tjalkens R. B. (2019). Genetic suppression of IKK2/NF-κB in astrocytes inhibits neuroinflammation and reduces neuronal loss in the MPTP-probenecid model of Parkinson's disease. Neurobiol. Dis. 127, 193–209. doi: 10.1016/j.nbd.2019.02.020, PMID: 30818064 PMC6588478

[ref65] Kluss J. H. Mamais A. Cookson M. R. (2019). LRRK2 links genetic and sporadic Parkinson's disease. Biochem. Soc. Trans. 47, 651–661. doi: 10.1042/BST20180462, PMID: 30837320 PMC6563926

[ref66] Kobayashi Y. Imamura R. Koyama Y. Kondo M. Kobayashi H. Nonomura N.. (2020). Renoprotective and neuroprotective effects of enteric hydrogen generation from Si-based agent. Sci. Rep. 10:5859. doi: 10.1038/s41598-020-62755-9, PMID: 32246095 PMC7125117

[ref67] Kostrzewa R. M. Jacobowitz D. M. (1974). Pharmacological actions of 6-hydroxydopamine. Pharmacol. Rev. 26, 199–288. doi: 10.1016/S0031-6997(25)06677-3 4376244

[ref68] Kubota M. Kobayashi N. Sugizaki T. Shimoda M. Kawahara M. Tanaka K. I. (2020). Carnosine suppresses neuronal cell death and inflammation induced by 6-hydroxydopamine in an in vitro model of Parkinson's disease. PLoS One 15:e0240448. doi: 10.1371/journal.pone.0240448, PMID: 33052927 PMC7556511

[ref69] Kwon H. S. Koh S. H. (2020). Neuroinflammation in neurodegenerative disorders: the roles of microglia and astrocytes. Transl. Neurodegener. 9:42. doi: 10.1186/s40035-020-00221-2, PMID: 33239064 PMC7689983

[ref70] Lee D. Choi J. I. (2021). Hydrogen-rich water improves cognitive ability and induces Antioxidative, Antiapoptotic, and anti-inflammatory effects in an acute ischemia-reperfusion injury mouse model. Biomed. Res. Int. 2021:9956938. doi: 10.1155/2021/9956938, PMID: 34746315 PMC8566066

[ref71] Li J. Liu H. Wang X. Xia Y. Huang J. Wang T.. (2022). Melatonin ameliorates Parkinson's disease via regulating microglia polarization in a RORα-dependent pathway. NPJ Parkinson's Dis. 8:90. doi: 10.1038/s41531-022-00352-5 35803929 PMC9270337

[ref72] Li W. Yang S. Yu F. Y. Zhao Y. Sun Z. M. An J. R.. (2018). Hydrogen ameliorates chronic intermittent hypoxia-induced neurocognitive impairment via inhibiting oxidative stress. Brain Res. Bull. 143, 225–233. doi: 10.1016/j.brainresbull.2018.09.012, PMID: 30243887

[ref73] Lin C. P. Chuang W. C. Lu F. J. Chen C. Y. (2017). Anti-oxidant and anti-inflammatory effects of hydrogen-rich water alleviate ethanol-induced fatty liver in mice. World J. Gastroenterol. 23, 4920–4934. doi: 10.3748/wjg.v23.i27.4920, PMID: 28785146 PMC5526762

[ref74] Liu C. Kurokawa R. Fujino M. Hirano S. Sato B. Li X. K. (2014). Estimation of the hydrogen concentration in rat tissue using an airtight tube following the administration of hydrogen via various routes. Sci. Rep. 4:5485. doi: 10.1038/srep05485, PMID: 24975958 PMC4074787

[ref75] Liu W. Lim K. L. Tan E. K. (2022). Intestine-derived α-synuclein initiates and aggravates pathogenesis of Parkinson's disease in Drosophila. Trans. Neurodegen. 11:44. doi: 10.1186/s40035-022-00318-w, PMID: 36253844 PMC9575256

[ref76] Liu L. Yang C. Qiu T. Shen X. Liu B. Qi X.. (2021). Hydrogen alleviates acute lung injury induced by limb ischaemia/reperfusion in mice. Life Sci. 279:119659. doi: 10.1016/j.lfs.2021.119659, PMID: 34052293

[ref77] Mahul-Mellier A. L. Burtscher J. Maharjan N. Weerens L. Croisier M. Kuttler F.. (2020). The process of Lewy body formation, rather than simply α-synuclein fibrillization, is one of the major drivers of neurodegeneration. Proc. Natl. Acad. Sci. USA 117, 4971–4982. doi: 10.1073/pnas.1913904117, PMID: 32075919 PMC7060668

[ref78] Man Anh H. Linh D. M. My Dung V. Thao T. P. (2019). Evaluating dose- and time-dependent effects of Vitamin C treatment on a Parkinson's disease Fly model. Parkinsons Dis. 2019, 1–14. doi: 10.1155/2019/9720546, PMID: 30719278 PMC6334328

[ref79] Matsumoto A. Yamafuji M. Tachibana T. Nakabeppu Y. Noda M. Nakaya H. (2013). Oral 'hydrogen water' induces neuroprotective ghrelin secretion in mice. Sci. Rep. 3:3273. doi: 10.1038/srep03273, PMID: 24253616 PMC4070541

[ref80] McCarthy E. Barron A. Morales-Prieto N. Mazzocchi M. McCarthy C. M. Collins L. M.. (2022). Gene co-expression analysis of the human substantia Nigra identifies ZNHIT1 as an SNCA co-expressed gene that protects against α-Synuclein-induced impairments in neurite growth and mitochondrial dysfunction in SH-SY5Y cells. Mol. Neurobiol. 59, 2745–2757. doi: 10.1007/s12035-022-02768-9, PMID: 35175558 PMC9016026

[ref81] McCarty M. F. Lerner A. (2020). Nutraceuticals targeting generation and oxidant activity of Peroxynitrite may aid prevention and control of Parkinson's disease. Int. J. Mol. Sci. 21:624. doi: 10.3390/ijms21103624, PMID: 32455532 PMC7279222

[ref82] Meredith G. E. Totterdell S. Petroske E. Santa Cruz K. Callison R. C. Lau Y. S. (2002). Lysosomal malfunction accompanies alpha-synuclein aggregation in a progressive mouse model of Parkinson's disease. Brain Res. 956, 156–165. doi: 10.1016/S0006-8993(02)03514-X, PMID: 12426058

[ref83] Meyers A. K. Zhu X. (2020). The NLRP3 Inflammasome: metabolic regulation and contribution to Inflammaging. Cells 9:808. doi: 10.3390/cells9081808, PMID: 32751530 PMC7463618

[ref84] Michailidis M. Moraitou D. Tata D. A. Kalinderi K. Papamitsou T. Papaliagkas V. (2022). Alzheimer's disease as type 3 diabetes: common pathophysiological mechanisms between Alzheimer's disease and type 2 diabetes. Int. J. Mol. Sci. 23:687. doi: 10.3390/ijms23052687, PMID: 35269827 PMC8910482

[ref85] Migliore M. M. Ortiz R. Dye S. Campbell R. B. Amiji M. M. Waszczak B. L. (2014). Neurotrophic and neuroprotective efficacy of intranasal GDNF in a rat model of Parkinson's disease. Neuroscience 274, 11–23. doi: 10.1016/j.neuroscience.2014.05.019, PMID: 24845869

[ref86] Mizuno K. Sasaki A. T. Ebisu K. Tajima K. Kajimoto O. Nojima J.. (2017). Hydrogen-rich water for improvements of mood, anxiety, and autonomic nerve function in daily life. Med. Gas Res. 7, 247–255. doi: 10.4103/2045-9912.222448, PMID: 29497485 PMC5806445

[ref87] Moos T. Morgan E. H. (2004). The metabolism of neuronal iron and its pathogenic role in neurological disease: review. Ann. N. Y. Acad. Sci. 1012, 14–26. doi: 10.1196/annals.1306.002, PMID: 15105252

[ref88] Morris H. R. Spillantini M. G. Sue C. M. Williams-Gray C. H. (2024). The pathogenesis of Parkinson's disease. Lancet 403, 293–304. doi: 10.1016/S0140-6736(23)01478-2 38245249

[ref89] Murakami Y. Ito M. Ohsawa I. (2017). Molecular hydrogen protects against oxidative stress-induced SH-SY5Y neuroblastoma cell death through the process of mitohormesis. PLoS One 12:e0176992. doi: 10.1371/journal.pone.0176992, PMID: 28467497 PMC5415102

[ref90] Nagata K. Nakashima-Kamimura N. Mikami T. Ohsawa I. Ohta S. (2009). Consumption of molecular hydrogen prevents the stress-induced impairments in hippocampus-dependent learning tasks during chronic physical restraint in mice. Neuropsychopharmacology 34, 501–508. doi: 10.1038/npp.2008.95, PMID: 18563058

[ref91] Nagatani K. Nawashiro H. Takeuchi S. Tomura S. Otani N. Osada H.. (2013). Safety of intravenous administration of hydrogen-enriched fluid in patients with acute cerebral ischemia: initial clinical studies. Med. Gas Res. 3:13. doi: 10.1186/2045-9912-3-13, PMID: 23799921 PMC3694409

[ref92] Nagatsu T. Mogi M. Ichinose H. Togari A. (2000). Changes in cytokines and neurotrophins in Parkinson's disease. J. Neural Transm. Suppl. 60, 277–290. doi: 10.1007/978-3-7091-6301-6_19 11205147

[ref93] Nethathe G. D. Lipman J. Anderson R. Fuller P. J. Feldman C. (2024). Glucocorticoids with or without fludrocortisone in septic shock: a narrative review from a biochemical and molecular perspective. Br. J. Anaesth. 132, 53–65. doi: 10.1016/j.bja.2023.10.034, PMID: 38030548 PMC10797514

[ref94] Ning K. Liu W. W. Huang J. L. Lu H. T. Sun X. J. (2018). Effects of hydrogen on polarization of macrophages and microglia in a stroke model. Med. Gas Res. 8, 154–159. doi: 10.4103/2045-9912.248266, PMID: 30713668 PMC6352575

[ref95] Niu F. Xie W. Zhang W. Kawuki J. Yu X. Vitamin C. (2024). Vitamin E, β-carotene and risk of Parkinson's disease: a systematic review and dose-response meta-analysis of observational studies. Nutr. Neurosci. 27, 329–341. doi: 10.1080/1028415X.2023.2192561, PMID: 36961747

[ref96] Novosadova E. Dolotov O. Inozemtseva L. Novosadova L. Antonov S. Shimchenko D.. (2022). Influence of N-Arachidonoyl dopamine and N-Docosahexaenoyl dopamine on the expression of neurotrophic factors in neuronal differentiated cultures of human induced pluripotent stem cells under conditions of oxidative stress. Antioxidants 11:142. doi: 10.3390/antiox11010142 35052646 PMC8773408

[ref97] Ohsawa I. Nishimaki K. Yamagata K. Ishikawa M. Ohta S. (2008). Consumption of hydrogen water prevents atherosclerosis in apolipoprotein E knockout mice. Biochem. Biophys. Res. Commun. 377, 1195–1198. doi: 10.1016/j.bbrc.2008.10.156, PMID: 18996093

[ref98] Ono H. Nishijima Y. Adachi N. Tachibana S. Chitoku S. Mukaihara S.. (2011). Improved brain MRI indices in the acute brain stem infarct sites treated with hydroxyl radical scavengers, Edaravone and hydrogen, as compared to Edaravone alone A non-controlled study. Med. Gas Res. 1:12. doi: 10.1186/2045-9912-1-12, PMID: 22146068 PMC3231971

[ref99] Oracz J. Zyzelewicz D. (2019). In vitro antioxidant activity and FTIR characterization of high-molecular weight Melanoidin fractions from different types of cocoa beans. Antioxidants 8:560. doi: 10.3390/antiox8110560 31731784 PMC6912521

[ref100] Panicker N. Ge P. Dawson V. L. Dawson T. M. (2021). The cell biology of Parkinson's disease. J. Cell Biol. 220:95. doi: 10.1083/jcb.202012095, PMID: 33749710 PMC8103423

[ref101] Park S. Yoo J. E. Yeon G. B. Kim J. H. Lee J. S. Choi S. K.. (2021). Trophoblast glycoprotein is a new candidate gene for Parkinson's disease. NPJ Parkinsons Dis. 7:110. doi: 10.1038/s41531-021-00252-0, PMID: 34876581 PMC8651753

[ref102] Penders J. Kissner R. Koppenol W. H. (2014). ONOOH does not react with H2: potential beneficial effects of H2 as an antioxidant by selective reaction with hydroxyl radicals and peroxynitrite. Free Radic. Biol. Med. 75, 191–194. doi: 10.1016/j.freeradbiomed.2014.07.025 25086438

[ref103] Peng Z. Li X. J. Zhou Y. Zhang J. T. Zhu Q. Sun J. Q.. (2024). Hydrogen exerts neuroprotective effects after subarachnoid hemorrhage by attenuating neuronal ferroptosis and inhibiting neuroinflammation. Free Radic. Biol. Med. 215, 79–93. doi: 10.1016/j.freeradbiomed.2024.02.028, PMID: 38447853

[ref104] Pluta R. Januszewski S. Czuczwar S. J. (2022). Molecular hydrogen neuroprotection in post-ischemic neurodegeneration in the form of Alzheimer's disease Proteinopathy: underlying mechanisms and potential for clinical implementation-fantasy or reality? Int. J. Mol. Sci. 23:591. doi: 10.3390/ijms23126591, PMID: 35743035 PMC9224395

[ref105] Poewe W. Seppi K. Tanner C. M. Halliday G. M. Brundin P. Volkmann J.. (2017). Parkinson disease. Nat. Rev. Dis. Primers 3:17013. doi: 10.1038/nrdp.2017.13, PMID: 28332488

[ref106] Pozo Devoto V. M. Falzone T. L. (2017). Mitochondrial dynamics in Parkinson's disease: a role for α-synuclein? Dis. Model. Mech. 10, 1075–1087. doi: 10.1242/dmm.026294, PMID: 28883016 PMC5611962

[ref107] Qian L. Liu M. Shen J. Cen J. Zhao D. (2020). Hydrogen in patients with corticosteroid-refractory/dependent chronic graft-versus-host-disease: a single-arm, multicenter, open-label, phase 2 trial. Front. Immunol. 11:598359. doi: 10.3389/fimmu.2020.598359, PMID: 33324415 PMC7724019

[ref108] Qin C. Zhou L. Q. Ma X. T. Hu Z. W. Yang S. Chen M.. (2019). Dual functions of microglia in ischemic stroke. Neurosci. Bull. 35, 921–933. doi: 10.1007/s12264-019-00388-3, PMID: 31062335 PMC6754485

[ref109] Rangasamy S. B. Dasarathi S. Pahan P. Jana M. Pahan K. (2019). Low-dose aspirin upregulates tyrosine hydroxylase and increases dopamine production in dopaminergic neurons: implications for Parkinson's disease. J. Neuroimmune Pharmacol. 14, 173–187. doi: 10.1007/s11481-018-9808-3, PMID: 30187283 PMC6401361

[ref110] Rascol O. Perez-Lloret S. Ferreira J. J. (2015). New treatments for levodopa-induced motor complications. Mov. Disord. 30, 1451–1460. doi: 10.1002/mds.26362 26293004

[ref111] Ray S. Singh N. Kumar R. Patel K. Pandey S. Datta D.. (2020). α-Synuclein aggregation nucleates through liquid-liquid phase separation. Nat. Chem. 12, 705–716. doi: 10.1038/s41557-020-0465-9, PMID: 32514159

[ref112] Rayaprolu S. Bitarafan S. Santiago J. V. Betarbet R. Sunna S. Cheng L.. (2022). Cell type-specific biotin labeling in vivo resolves regional neuronal and astrocyte proteomic differences in mouse brain. Nat. Commun. 13:2927. doi: 10.1038/s41467-022-30623-x, PMID: 35614064 PMC9132937

[ref113] Rizig M. Bandres-Ciga S. Makarious M. B. Ojo O. Crea P. W. Abiodun O.. (2023). Genome-wide association identifies novel etiological insights associated with Parkinson's disease in African and African admixed populations. Medrxiv. 22, 1015–1025. doi: 10.1101/2023.05.05.23289529 PMC10593199 37633302

[ref114] Ruan H. Wang L. Wang J. Sun H. He X. Li W.. (2019). Sika deer antler protein against acetaminophen-induced oxidative stress and apoptosis in HK-2 cells via activating Nrf2/keap1/HO-1 pathway. J. Food Biochem. 43:e13067. doi: 10.1111/jfbc.13067, PMID: 31599006

[ref115] Sadaka A. H. Canuel J. Febo M. Johnson C. T. Bradshaw H. B. Ortiz R.. (2023). Effects of inhaled cannabis high in Δ9-THC or CBD on the aging brain: a translational MRI and behavioral study. Front. Aging Neurosci. 15:1055433. doi: 10.3389/fnagi.2023.1055433, PMID: 36819730 PMC9930474

[ref116] Sanches B. C. P. Rocha C. A. Martin Bedoya J. G. da Silva V. L. da Silva P. B. Fusco-Almeida A. M.. (2021). Rhamnolipid-based liposomes as promising Nano-carriers for enhancing the antibacterial activity of peptides derived from bacterial toxin-antitoxin systems. Int. J. Nanomedicine 16, 925–939. doi: 10.2147/IJN.S283400, PMID: 33603360 PMC7882795

[ref117] Santos K. G. D. Eckert C. T. Rossi E. D. Bariccatti R. A. Frigo E. P. Lindino C. A.. (2017). Hydrogen production in the electrolysis of water in Brazil, a review. Renewable Sustainable Energy Reviews. 68, 563–571. doi: 10.1016/j.rser.2016.09.128

[ref118] Sawner A. S. Ray S. Yadav P. Mukherjee S. Panigrahi R. Poudyal M.. (2021). Modulating α-Synuclein liquid-liquid phase separation. Biochemistry 60, 3676–3696. doi: 10.1021/acs.biochem.1c00434, PMID: 34431665

[ref119] Schmidt N. Ferger B. (2001). Neurochemical findings in the MPTP model of Parkinson's disease. J. Neural Transm. 108, 1263–1282. doi: 10.1007/s007020100004 11768626

[ref120] Schober A. (2004). Classic toxin-induced animal models of Parkinson's disease: 6-OHDA and MPTP. Cell Tissue Res. 318, 215–224. doi: 10.1007/s00441-004-0938-y, PMID: 15503155

[ref121] Shahmoradian S. H. Lewis A. J. Genoud C. Hench J. Moors T. E. Navarro P. P.. (2019). Lewy pathology in Parkinson's disease consists of crowded organelles and lipid membranes. Nat. Neurosci. 22, 1099–1109. doi: 10.1038/s41593-019-0423-2, PMID: 31235907

[ref122] Sharma S. Narang J. K. Ali J. Baboota S. (2016). Synergistic antioxidant action of vitamin E and rutin SNEDDS in ameliorating oxidative stress in a Parkinson's disease model. Nanotechnology 27:375101. doi: 10.1088/0957-4484/27/37/375101, PMID: 27491690

[ref123] Shen X. Wong-Yu I. S. Mak M. K. (2016). Effects of exercise on falls, balance, and gait ability in Parkinson's disease: a Meta-analysis. Neurorehabil. Neural Repair 30, 512–527. doi: 10.1177/1545968315613447, PMID: 26493731

[ref124] Shin Y. J. Kim Y. J. Lee J. E. Kim Y. S. Lee J. W. Kim H.. (2023). Uric acid regulates α-synuclein transmission in parkinsonian models. Front. Aging Neurosci. 15:1117491. doi: 10.3389/fnagi.2023.1117491, PMID: 37711993 PMC10497982

[ref125] Sim M. Kim C. S. Shon W. J. Lee Y. K. Choi E. Y. Shin D. M. (2020). Hydrogen-rich water reduces inflammatory responses and prevents apoptosis of peripheral blood cells in healthy adults: a randomized, double-blind, controlled trial. Sci. Rep. 10:12130. doi: 10.1038/s41598-020-68930-2, PMID: 32699287 PMC7376192

[ref126] Soni D. Jamwal S. Chawla R. Singh S. Singh D. Singh T. G.. (2024). Nutraceuticals unveiled a multifaceted neuroprotective mechanisms for Parkinson’s disease: elixir for the brain. Food Rev. Intl. 40, 3079–3102. doi: 10.1080/87559129.2024.2337766, PMID: 40101104

[ref127] Soni D. Kumar P. (2022). GSK-3β-mediated regulation of Nrf2/HO-1 signaling as a new therapeutic approach in the treatment of movement disorders. Pharmacol. Rep. 74, 557–569. doi: 10.1007/s43440-022-00390-z, PMID: 35882765

[ref128] Soni D. Upadhayay S. Dhureja M. Arthur R. Kumar P. (2024). Crosstalk between gut-brain axis: unveiling the mysteries of gut ROS in progression of Parkinson's disease. Inflammopharmacology 32, 2921–2941. doi: 10.1007/s10787-024-01510-2, PMID: 38992324

[ref129] Taguchi T. Ikuno M. Hondo M. Parajuli L. K. Taguchi K. Ueda J.. (2020). α-Synuclein BAC transgenic mice exhibit RBD-like behaviour and hyposmia: a prodromal Parkinson's disease model. Brain 143, 249–265. doi: 10.1093/brain/awz380, PMID: 31816026

[ref130] Takeuchi S. Kumagai K. Toyooka T. Otani N. Wada K. Mori K. (2021). Intravenous hydrogen therapy with intracisternal magnesium sulfate infusion in severe aneurysmal subarachnoid hemorrhage. Stroke 52, 20–27. doi: 10.1161/STROKEAHA.120.031260, PMID: 33349011

[ref131] Tanifum E. A. Dasgupta I. Srivastava M. Bhavane R. C. Sun L. Berridge J.. (2012). Intravenous delivery of targeted liposomes to amyloid-β pathology in APP/PSEN1 transgenic mice. PLoS One 7:e48515. doi: 10.1371/journal.pone.0048515, PMID: 23119043 PMC3485335

[ref132] Tassone A. Meringolo M. Ponterio G. Bonsi P. Schirinzi T. Martella G. (2023). Mitochondrial bioenergy in neurodegenerative disease: Huntington and Parkinson. Int. J. Mol. Sci. 24:221. doi: 10.3390/ijms24087221, PMID: 37108382 PMC10138549

[ref133] Tichelaar J. G. Sayalı C. Helmich R. C. Cools R. (2023). Impulse control disorder in Parkinson's disease is associated with abnormal frontal value signalling. Brain 146, 3676–3689. doi: 10.1093/brain/awad162, PMID: 37192341 PMC10473575

[ref134] Topal G. R. Mészáros M. Porkoláb G. Szecskó A. Polgár T. F. Siklós L.. (2020). ApoE-targeting increases the transfer of solid lipid nanoparticles with donepezil cargo across a culture model of the blood-brain barrier. Pharmaceutics 13:38. doi: 10.3390/pharmaceutics13010038, PMID: 33383743 PMC7824445

[ref135] van Rumund A. Pavelka L. Esselink R. A. J. Geurtz B. P. M. Wevers R. A. Mollenhauer B.. (2021). Peripheral decarboxylase inhibitors paradoxically induce aromatic L-amino acid decarboxylase. NPJ Parkinsons Dis. 7:29. doi: 10.1038/s41531-021-00172-z, PMID: 33741988 PMC7979935

[ref136] Veys L. Devroye J. Lefevere E. Cools L. Vandenabeele M. De Groef L. (2021). Characterizing the retinal phenotype of the Thy1-h[A30P]α-syn mouse model of Parkinson's disease. Front. Neurosci. 15:726476. doi: 10.3389/fnins.2021.726476, PMID: 34557068 PMC8452874

[ref137] Wang J. Cheng Q. Fang J. Ding H. Liu H. Fang X.. (2021). A preliminary study on the effect of hydrogen gas on alleviating early CCl(4)-induced chronic liver injury in rats. Antioxidants 10:1933. doi: 10.3390/antiox10121933 34943036 PMC8750042

[ref138] Wang Y. A. van Sluijs L. Nie Y. Sterken M. G. Harvey S. C. Kammenga J. E. (2020). Genetic variation in complex traits in transgenic α-Synuclein strains of *Caenorhabditis elegans*. Genes 11:78. doi: 10.3390/genes11070778, PMID: 32664512 PMC7397059

[ref139] Wang X. Y. Xu C. C. (2021). Clinical study of hydrogen-rich water in the treatment of 64 cases of Parkinson's disease. Naval Med. J. 42, 108–109.

[ref140] Wang P. Zhao M. Chen Z. Wu G. Fujino M. Zhang C.. (2020). Hydrogen gas attenuates hypoxic-ischemic brain injury via regulation of the MAPK/HO-1/PGC-1a pathway in neonatal rats. Oxidative Med. Cell. Longev. 2020, 1–16. doi: 10.1155/2020/6978784, PMID: 32104537 PMC7040418

[ref141] Wolff A. Schumacher N. U. Pürner D. Machetanz G. Demleitner A. F. Feneberg E.. (2023). Parkinson's disease therapy: what lies ahead? J. Neural Trans. 130, 793–820. doi: 10.1007/s00702-023-02641-6 PMC10199869 37147404

[ref142] Wu L. Liu M. Liang J. Li N. Yang D. Cai J.. (2021). Ferroptosis as a new mechanism in Parkinson's disease therapy using traditional Chinese Medicine. Front. Pharmacol. 12:659584. doi: 10.3389/fphar.2021.659584, PMID: 34163356 PMC8215498

[ref143] Wu M. Yang Y. Wang M. Zeng F. Li Q. Liu W.. (2018). Exogenous pancreatic Kallikrein improves diabetic cardiomyopathy in Streptozotocin-induced diabetes. Front. Pharmacol. 9:855. doi: 10.3389/fphar.2018.00855, PMID: 30131697 PMC6091235

[ref144] Xia C. F. Boado R. J. Zhang Y. Chu C. Pardridge W. M. (2008). Intravenous glial-derived neurotrophic factor gene therapy of experimental Parkinson's disease with Trojan horse liposomes and a tyrosine hydroxylase promoter. J. Gene Med. 10, 306–315. doi: 10.1002/jgm.1152, PMID: 18085726

[ref145] Xie W. Gao J. Jiang R. Liu X. Lai F. Tang Y.. (2020). Twice subacute MPTP administrations induced time-dependent dopaminergic neurodegeneration and inflammation in midbrain and ileum, as well as gut microbiota disorders in PD mice. Neurotoxicology 76, 200–212. doi: 10.1016/j.neuro.2019.11.009, PMID: 31790727

[ref146] Xie L. Zhu Q. Zhang G. Ye K. Zou C. Prezhdo O. V.. (2020). Tunable hydrogen doping of metal oxide semiconductors with acid-metal treatment at ambient conditions. J. Am. Chem. Soc. 142, 4136–4140. doi: 10.1021/jacs.0c00561, PMID: 32081005

[ref147] Yang J. T. Kuo Y. C. Lee K. C. De S. Chen Y. Y. (2025). Resveratrol and ceftriaxone encapsulated in hybrid nanoparticles to prevent dopaminergic neurons from degeneration for Parkinson's disease treatment. Biomater. Adv. 166:214065. doi: 10.1016/j.bioadv.2024.214065, PMID: 39426178

[ref148] Yang L. Li D. Chen S. (2016). Hydrogen water reduces NSE, IL-6, and TNF-α levels in hypoxic-ischemic encephalopathy. Open Med. 11, 399–406. doi: 10.1515/med-2016-0072, PMID: 28352827 PMC5329859

[ref149] Yang Y. Liu P. Y. Bao W. Chen S. J. Wu F. S. Zhu P. Y. (2020). Hydrogen inhibits endometrial cancer growth via a ROS/NLRP3/caspase-1/GSDMD-mediated pyroptotic pathway. BMC Cancer 20:28. doi: 10.1186/s12885-019-6491-6, PMID: 31924176 PMC6954594

[ref150] Yang C. Wang W. Deng P. Li C. Zhao L. Gao H. (2021). Fibroblast growth factor 21 modulates microglial polarization that attenuates neurodegeneration in mice and cellular models of Parkinson's disease. Front. Aging Neurosci. 13:778527. doi: 10.3389/fnagi.2021.778527, PMID: 35002679 PMC8727910

[ref151] Yang Y. Zhu Y. Xi X. (2018). Anti-inflammatory and antitumor action of hydrogen via reactive oxygen species. Oncol. Lett. 16, 2771–2776. doi: 10.3892/ol.2018.9023, PMID: 30127861 PMC6096066

[ref152] Yoritaka A. Abe T. Ohtsuka C. Maeda T. Hirayama M. Watanabe H.. (2016). A randomized double-blind multi-center trial of hydrogen water for Parkinson's disease: protocol and baseline characteristics. BMC Neurol. 16:66. doi: 10.1186/s12883-016-0589-0, PMID: 27176725 PMC4865993

[ref153] Yoritaka A. Abe T. Ohtsuka C. Maeda T. Hirayama M. Watanabe H.. (2017). Erratum to: a randomized double-blind multi-center trial of hydrogen water for Parkinson's disease: protocol and baseline characteristics. BMC Neurol. 17:35. doi: 10.1186/s12883-017-0817-2, PMID: 28219346 PMC5322784

[ref154] Yoritaka A. Kobayashi Y. Hayashi T. Saiki S. Hattori N. (2021). Randomized double-blind placebo-controlled trial of hydrogen inhalation for Parkinson's disease: a pilot study. Neurol. Sci. 42, 4767–4770. doi: 10.1007/s10072-021-05489-4, PMID: 34319514 PMC8519836

[ref155] Yoritaka A. Ohtsuka C. Maeda T. Hirayama M. Abe T. Watanabe H.. (2018). Randomized, double-blind, multicenter trial of hydrogen water for Parkinson's disease. Mov. Disord. 33, 1505–1507. doi: 10.1002/mds.27472, PMID: 30207619

[ref156] Yoritaka A. Takanashi M. Hirayama M. Nakahara T. Ohta S. Hattori N. (2013). Pilot study of H₂ therapy in Parkinson's disease: a randomized double-blind placebo-controlled trial. Mov. Disord. 28, 836–839. doi: 10.1002/mds.25375, PMID: 23400965

[ref157] Yoshii Y. Inoue T. Uemura Y. Iwasaki Y. Yada T. Nakabeppu Y.. (2017). Complexity of stomach-brain interaction induced by molecular hydrogen in Parkinson's disease model mice. Neurochem. Res. 42, 2658–2665. doi: 10.1007/s11064-017-2281-1, PMID: 28462451

[ref158] Zhang Y. G. Sheng Q. S. Wang Z. J. Lv L. I. Zhao W. Chen J. M.. (2015). Hydrogen-rich saline promotes motor functional recovery following peripheral nerve autografting in rats. Exp. Ther. Med. 10, 727–732. doi: 10.3892/etm.2015.2518 26622383 PMC4508974

[ref159] Zhang Z. Sun X. Wang K. Yu Y. Zhang L. Zhang K.. (2021). Hydrogen-saturated saline mediated neuroprotection through autophagy via PI3K/AKT/mTOR pathway in early and medium stages of rotenone-induced Parkinson's disease rats. Brain Res. Bull. 172, 1–13. doi: 10.1016/j.brainresbull.2021.04.003, PMID: 33838212

[ref160] Zhang Y. X. Xu J. T. You X. C. Wang C. Zhou K. W. Li P.. (2016). Inhibitory effects of hydrogen on proliferation and migration of vascular smooth muscle cells via Down-regulation of mitogen/activated protein kinase and Ezrin/radixin/Moesin signaling pathways. Chin. J. Physiol. 59, 46–55. doi: 10.4077/CJP.2016.BAE365 26875562

[ref161] Zhuang Z. Sun X. J. Zhang X. Liu H. D. You W. C. Ma C. Y.. (2013). Nuclear factor-κB/Bcl-XL pathway is involved in the protective effect of hydrogen-rich saline on the brain following experimental subarachnoid hemorrhage in rabbits. J. Neurosci. Res. 91, 1599–1608. doi: 10.1002/jnr.23281 24105634

[ref162] Zhuang X. Yu Y. Jiang Y. Zhao S. Wang Y. Su L.. (2020). Molecular hydrogen attenuates sepsis-induced neuroinflammation through regulation of microglia polarization through an mTOR-autophagy-dependent pathway. Int. Immunopharmacol. 81:106287. doi: 10.1016/j.intimp.2020.106287, PMID: 32058932

